# Applicability of mitotic figure counting by deep learning: a development and pan‐cancer validation study

**DOI:** 10.1002/2211-5463.70210

**Published:** 2026-02-12

**Authors:** Joakim Kalsnes, Maria X. Isaksen, Frida Julbø, Manohar Pradhan, Andreas Kleppe, Sepp De Raedt, Ole‐Johan Skrede, Turid Torheim, John Arne Nesheim, Hans Martin Mohn, Hanne A. Askautrud, Karolina Cyll, Wanja Kildal, Emma Rewcastle, Melinda Lillesand, Vebjørn Kvikstad, Emiel Janssen, Robert Jones, Odd Terje Brustugun, Bjørn Brennhovd, Erik Skaaheim Haug, Lill‐Tove Rasmussen Busund, Elin Richardsen, Sigve Andersen, Tom Dønnem, Kristina Lindemann, Gunnar Kristensen, Neil A. Shepherd, Marco Novelli, Knut Liestøl, David Kerr, Håvard E. Danielsen, Tarjei S. Hveem

**Affiliations:** ^1^ Institute for Cancer Genetics and Informatics Oslo University Hospital Norway; ^2^ Department of Informatics University of Oslo Norway; ^3^ Centre for Research‐based Innovation Visual Intelligence UiT The Arctic University of Norway Tromsø Norway; ^4^ Department of Pathology Stavanger University Hospital Norway; ^5^ Department of Chemistry, Bioscience and Environmental Engineering Stavanger University Norway; ^6^ Department of Hepatobiliary Surgery Liverpool University Hospitals NHS Foundation Trust UK; ^7^ Department of Molecular and Clinical Cancer Medicine University of Liverpool UK; ^8^ Section of Oncology Vestre Viken Hospital Trust Drammen Norway; ^9^ Institute of Clinical Medicine University of Oslo Norway; ^10^ Department of Urology Oslo University Hospital Norway; ^11^ Department of Urology Vestfold Hospital Trust Tønsberg Norway; ^12^ Department of Medical Biology, UiT The Arctic University of Norway Tromsø Norway; ^13^ Department of Clinical Pathology University Hospital of North Norway Tromsø Norway; ^14^ Department of Oncology University Hospital of North Norway Tromso Norway; ^15^ Institute of Clinical Medicine, UiT The Arctic University of Norway Tromso Norway; ^16^ Section for Gynecological Oncology, Department of Surgical Oncology Oslo University Hospital Norway; ^17^ Gloucestershire Cellular Pathology Laboratory Cheltenham General Hospital UK; ^18^ Research Department of Pathology University College London Medical School UK; ^19^ Nuffield Division of Clinical Laboratory Sciences University of Oxford UK

**Keywords:** cancer, deep learning, digital pathology, mitotic figure count, prognosis, validation study

## Abstract

Mitotic figure counting is an established measure of cell proliferation that is included in grading systems. We developed a deep learning method for mitotic figure counting and evaluated its prognostic impact in multiple external validation datasets. The deep learning method was trained in whole slide images of tissue sections stained with haematoxylin and eosin from a publicly available breast cancer dataset where mitotic figures have been annotated by expert pathologists. The final model was externally validated according to a protocol with predefined analyses of 14 571 patient samples from 13 patient cohorts from seven different cancer types. The predefined primary analysis was univariable Cox survival analysis of the number of mitotic figures detected per mm^2^. Automatic mitotic figure counting correlated well with known proliferation rates, and patients with more mitotic figures per mm^2^ had significantly worse patient outcome in all the studied cancer types except colorectal cancer. This study demonstrates the practical potential of automated, deep learning‐based mitotic figure counting, both by automating pathology work and by suggesting expanded use in more cancer types, such as prostate cancer.

## Graphical abstract

In this study, we developed a deep learning method for mitotic figure counting in H&E‐stained whole‐slide images and evaluated its prognostic impact in 13 external validation cohorts from seven different cancer types. Patients with more mitotic figures per mm^2^ had significantly worse patient outcome in all the studied cancer types except colorectal cancer.

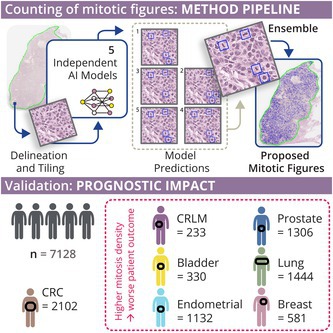

**Abbreviations**
CI: confidence intervals
CRC: colorectal cancer
CRLM: colorectal cancer with liver metastasis
FFPE: formalin‐fixed and paraffin‐embedded
HE: haematoxylin and Eosin
HPF: high power fields
HR: hazard ratio
ICGI: Institute for Cancer Genetics and Informatics
IQR: interquartile range
NSCLC: nonsmall cell lung cancer
OUS: Oslo University Hospital
PCa: prostate cancer
PCNA: proliferating cell nuclear antigen
PHH3: phosphohistone H3
PIECES: protocol Items for external cohort evaluation of a deep learning System
PSA: prostate‐specific antigen
SUH: Stavanger University Hospital
TUPAC16: Tumour Proliferation Assessment Challenge 2016
UNN: University Hospital of North Norway
VHT: Vestfold Hospital Trust
WSI: whole slide image

## Introduction

Boveri postulated that cancer is a problem of cell proliferation [1]. The proliferation rate of cancer cells has been used to characterise tumour aggressiveness for decades. Common markers of cellular proliferation include Ki67, proliferating cell nuclear antigen (PCNA), phosphohistone H3 (PHH3) and mitotic figure counting [2]. The density of mitotic figures is a prognostic marker in several cancer types and is one of three components in the Nottingham grading system used for breast cancer [3]. Pathologists count mitotic figures in a fixed‐size region of the tumour, and the findings are translated into risk categories. The method is slow, and the interobserver variation is substantial when a strict protocol is not followed [4, 5]. Table S1 provides an overview of applications of mitotic figure counting in the clinic.

With the advent of computerised analysis of scanned histopathological tissue sections, automatic identification of mitotic figures is an obvious target. Challenges have been arranged in this field where the participants are provided a training dataset where pathologists have annotated mitotic figures. The contestants develop methods for the automatic detection of mitotic figures and the submitted methods are eventually tested on unseen data to evaluate the ability to generalise [6, 7, 8]. However, despite reports with promising solutions [9, 10, 11], studies showing the applicability of automatic mitotic figure assessment across large cancer patient datasets have been limited. Stathonikos *et al*. [12] demonstrated that, within a large external breast cancer dataset, an automated mitotic count algorithm performs comparably to traditional manual counting in predicting overall survival.

In this work, we evaluate the applicability of automatic mitotic figure assessment across cancer types and determine its prognostic value using more than 7000 patients from eight different cancer types: prostate cancer, bladder cancer, breast cancer, endometrial cancer, uterine sarcomas, lung cancer, colorectal cancer and liver metastases.

## Methods

### Development dataset

Publicly available images of breast cancer specimens with annotated mitotic figures from the Tumour Proliferation Assessment Challenge 2016 (TUPAC16) challenge [7] were used to train and tune the method. The dataset consisted of one or more variably sized regions (median 2.0 mm^2^ [interquartile range (IQR) 2.0–2.5 mm^2^]) from scanned haematoxylin and eosin (HE)‐sections from 73 patients from three pathology centres in the Netherlands (see ‘Development dataset’ in Appendix S1). All samples were scanned with 40× lens magnification and a spatial resolution of 0.25 μm/pixel. The mitotic figure annotation was based on a consensus of three pathologists [7]. The development set was split randomly on a scan level into a training subset constituting 70% of the samples, whereas the remaining 30% were used for tuning.

### Test and validation datasets

In order to (a) avoid overfitting and (b) provide nonbiased estimates of accuracy and generalisability, we have used (a) a test dataset during the development phase and (b) several validation datasets to evaluate the prognostic impact of the model. The prognostic impact in the test dataset was used to guide hyperparameter settings and model selection, whereas the validation datasets were analysed only once and according to the prespecified protocol (Supplementary Protocol in Appendix S1).

Whole slide images (WSIs) from a total population of uterine sarcomas in Norway diagnosed in the time period 1970–2000 [13] (Table S3) was included in the study as a test cohort to evaluate the prognostic performance of different model configurations (see ‘Test dataset’ in Appendix S1). In total, 372 patients had tumour samples and were eligible for analysis (Protocol Fig. 4 in Appendix S1).

The prognostic impact of the final method was validated according to the predefined protocol in 13 external cohorts from prostate, colorectal, lung, breast, bladder, liver metastasis and endometrial cancer (Tables 1, 2). Patient and sample inclusions and exclusions are described in the predefined protocol (see ‘External cohorts’ in Appendix S1; Protocol Figs 5–17), together with endpoints and variables to be included in multivariable analyses. Ethics approvals are listed in Table S2.

**Table 1:** Validation datasets for the evaluation of prognostic impact of automatic mitotic figure counting. CRC, colorectal cancer, CRLM, colorectal cancer with liver metastasis, NSCLC, nonsmall cell lung cancer, PCa, prostate cancer, PSA, prostate specific antigen.

Cancer type, cohort reference	Time period diagnosed	Hospital	Number of patients	Number of scanned slides	Endpoint primary analysis
PCa cohort 1 [25]	1987–2005	Oslo University Hospital, Oslo	253	750	Time to recurrence
PCa cohort 2 [26]	2001–2006	Oslo University Hospital, Oslo	259	777	Biochemical recurrence, PSA ≥ 0.4 ng·mL^−1^
PCa cohort 3	1999–2010	Vestfold Hospital Trust, Norway	326	970	Biochemical recurrence, PSA ≥ 0.4 ng·mL^−1^
PCa cohort 4 [27]	1995–2006	University hospitals of Northern Norway*	468	615	Biochemical recurrence, PSA ≥ 0.4 ng·mL^−1^
NSCLC	2006–2018	Oslo University Hospital	922	3485	Cancer‐specific survival
Stage I and II NSCLC [28, 29]	1990–2010	University hospital of North Norway and Nordland Hospital Trust	522	522	Cancer‐specific survival
Breast cancer cohort 1 [30]	1990–1998	Stavanger University Hospital	307	307	Time to distant recurrence
Breast cancer cohort 2 [31, 32]	2000–2004	Stavanger University Hospital	274	274	Time to distant recurrence
Endometrial cohort	2006–2017	Oslo University Hospital	1132	3028	Time to recurrence
Bladder cohort [33, 34]	1992–2010	Stavanger University Hospital	330	330	Time to stage progression
CRLM	2010–2015	Liverpool University Hospitals, England	233	1388	Overall survival
Stage I‐III CRC [35, 36]	1988–1997	The Gloucester CRC study, Cheltenham, England	978	978	Cancer‐specific survival
Stage II‐III CRC [37]	1994–2003	QUASAR 2 trial	1124	1147	Cancer‐specific survival

* St. Olav Hospital/Trondheim University Hospital, Nordlandssykehuset Bodo and the University Hospital of Northern Norway.

**Table 2:** Overview of predefined primary and secondary analysis.

	Variable	Endpoint	Analysis
Primary	Mitotic figures per mm^2^ (log‐transformed)	Specified for each cancer type in protocol	Univariable Cox regression
Secondary	Mitotic figures per mm^2^ (log‐transformed) with established prognostic markers	Same as primary	Multivariable Cox regression
	Mitotic figures per mm^2^ categorised by quartiles	Same as primary	Univariable Cox regression
	Mitotic figures per mm^2^ (log‐transformed)	Different than primary. Specified in protocol	Univariable Cox regression

Slides were made by staining thin formalin‐fixed and paraffin‐embedded (FFPE) tissue block sections with HE. The samples from Stavanger University Hospital and University hospitals of Northern Norway were prepared at the local institutions; the other samples were prepared at the Institute for Cancer Genetics and Informatics (ICGI). The samples were scanned on an Aperio AT2 scanner (Leica Biosystems, Germany) at the highest available resolution, referred to as 40× magnification by the manufacturer. The tumour regions were digitally annotated by a pathologist (M.P.).

### Model training

An ensemble of five Mask R‐CNN neural networks was trained to detect mitotic figures in the development dataset as described in the protocol (see ‘System’ in Appendix S1, Fig. S1). The method uses digital scans from HE‐stained tissue sections with manual tumour annotations from which tile images of size 1024 × 1024 pixels are extracted. The extracted tile images were normalised with the method described by Macenko *et al*. [14]. The tile images were input to the mitosis detector and a list of mitotic figure predictions was output, where each prediction has a score assigned to it, representing the probability of belonging to the mitotic figure class.

**Fig. 1:**
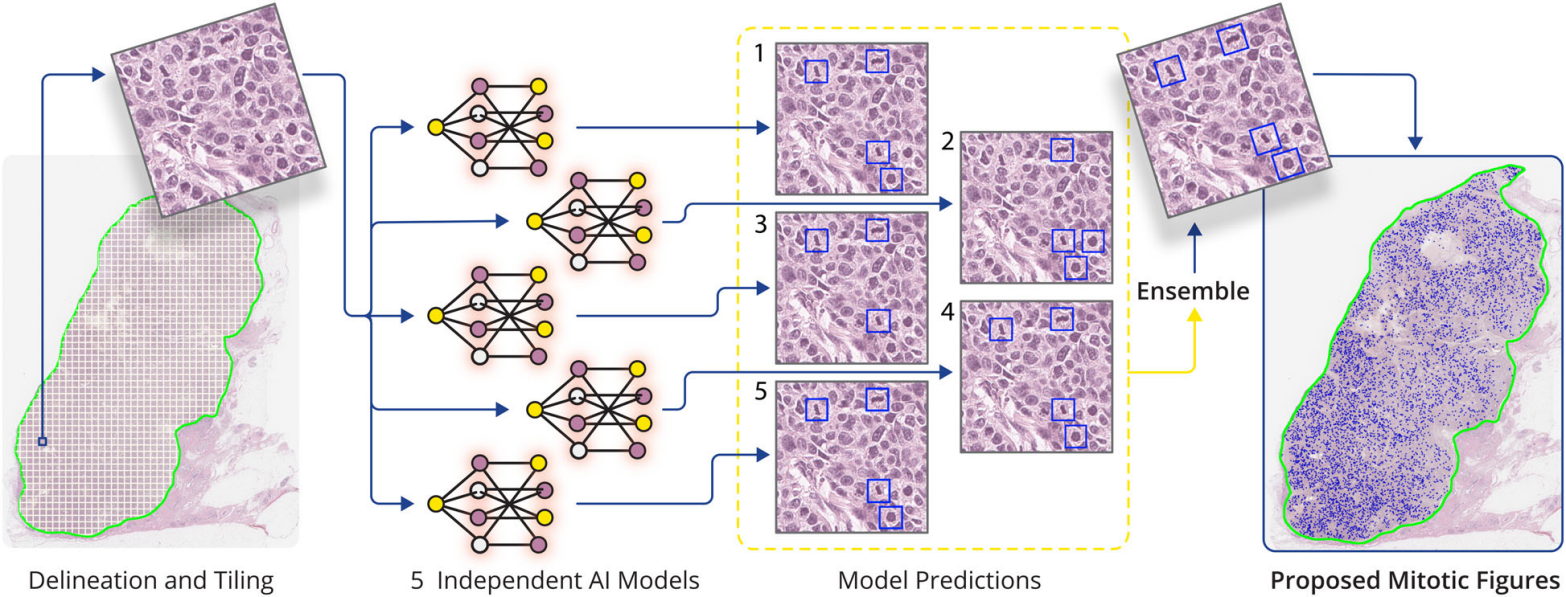
Pipeline of automatic mitotic figure counting. A whole‐slide image is segmented for tumour, and the segmented region is split into nonoverlapping image tiles of size 1024 × 1024 pixels at 40× resolution. The extracted tiles are analysed by the five independent trained deep learning models for detection of mitotic figures. Proposed mitotic figures are given a score between 0 and 1, corresponding to the model's certainty of a true detection. The output from the five models is combined into an ensemble prediction by calculating the average score for each proposed mitotic figure and filtering out detections with an average score below 0.4. Lastly, the number of proposed mitotic figures for the whole tumour (marked by blue dots) divided by total tumour area (mm^2^) is reported.

The five individual models were combined in an ensemble model, with an average score calculated for each detection. Detections with an average score below a set threshold were filtered out (Fig. S2). Each sample in the dataset was represented by the number of detected mitotic figures divided by the total tumour area (mm^2^). An overview of how the model is applied in digital HE‐stained tissue sections is shown in Fig. 1. We trained ensemble models with different configurations for data augmentation. The ensemble models were evaluated in the test dataset using concordance index in analysis of overall survival as a measure of accuracy (Table S4) [15]. The analysis in the test dataset was univariable Cox survival analysis of the number of mitotic figures detected per mm^2^, represented as a log_2_‐transformed continuous variable. This was in line with the primary analysis used for the validation datasets. The evaluated models demonstrated a similar concordance index level; for generalizability we thus selected the ensemble model with the highest level of augmentation.

**Fig. 2:**
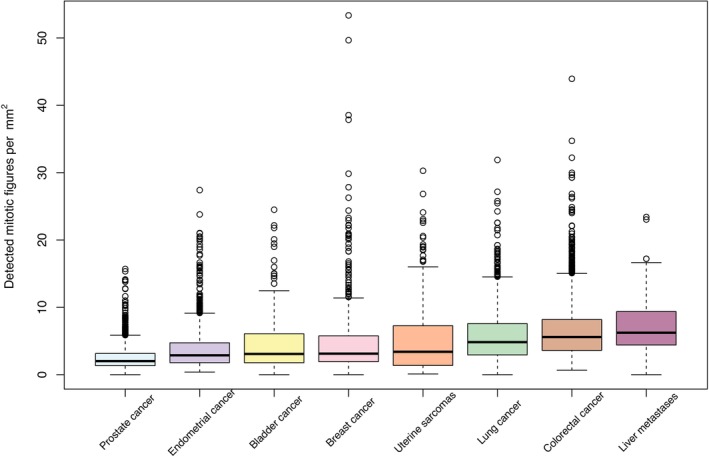
Detected mitotic figures per mm^2^ in the studied cancer types. Box plot with the number of detected mitotic figures per mm^2^ in the studied cancer types. Cancer types from both the test dataset (uterine sarcoma, number of patients (*N*) = 372) and the validation datasets (prostate cancer, *N* = 1306; endometrial cancer, *N* = 1132; bladder cancer, *N* = 330; breast cancer, *N* = 581; lung cancer, *N* = 1444; colorectal cancer, *N* = 2102; colorectal cancer with liver metastasis, *N* = 233) are included. The boxes visualise the interquartile range, and the horizontal line represents the median. The whiskers extend to the minimum and maximum values within 1.5 times the interquartile range from the first and third quartiles, respectively.

**Fig. 3:**
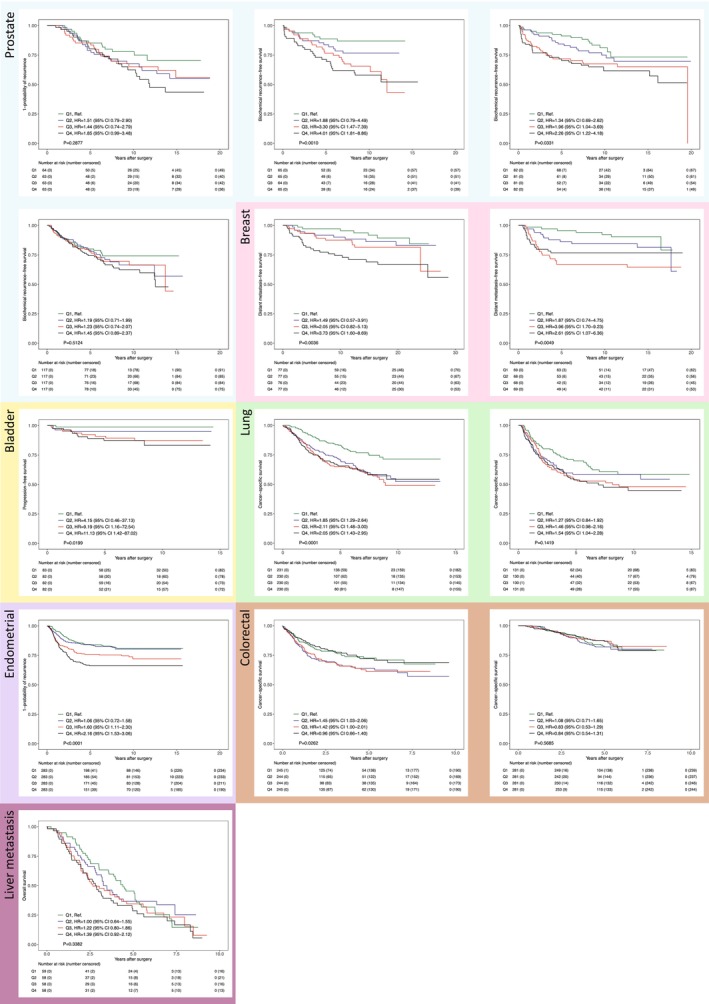
Kaplan–Meier survival analyses of mitotic figures per mm^2^. Kaplan–Meier survival analysis for each of the 13 external validation sets with the endpoint specified in the *y*‐axis for each dataset. The *P*‐values were calculated using the Mantel–Cox log‐rank test.

In postprocessing, detections along the edges of the tiles were handled to avoid duplicate detections of the same mitotic figure on neighbouring tiles (see ‘Edge detections’ in Appendix S1; Protocol Figs 1–3).

### Comparison with established pathology practice

In clinical breast cancer pathology, mitotic figures are counted manually in 10 high power fields (HPF), corresponding to about 1–3 mm^2^, selected as a consecutive region with high cellular proliferation. Mitotic figures were counted automatically in circle shaped regions of size 2 mm^2^ in *post hoc* analyses to mimic the mitotic figure counting in hotspots with high cellular proliferation performed by pathologists. The region with the highest mitotic figure density was used to represent the sample (as mitotic figures per mm^2^). The analyses were performed in the uterine sarcoma and the breast cancer datasets.

### Statistical analysis

Primary and secondary analyses were specified before the evaluation in the validation datasets and are described in the protocol written in accordance with *Protocol Items for External Cohort Evaluation of a deep learning System (PIECES)* [16]. Each validation dataset was analysed separately. The predefined primary analysis was univariable Cox survival analysis of the number of mitotic figures detected per mm^2^, represented as a log_2_‐transformed continuous variable. The survival endpoint for each validation dataset was predefined and is indicated in Table 1. Hazard ratios (HR) are given with 95% confidence intervals (CI); *P*‐values were calculated using the Wald *χ* ^2^ test. The number of mitotic figures per mm^2^ was evaluated in predefined multivariable Cox models as secondary and post hoc analyses. *P*‐values in the multivariable analyses were calculated using the Wald *χ* ^2^ test. Patients with at least one missing value were excluded. The Mantel–Cox log‐rank test was used to test whether the categorised mitotic figure marker predicted survival outcome in univariable analyses. A two‐sided *P*‐value of less than 0.05 was considered statistically significant. Statistical analyses were done with R version 3.5.2.

## Results

The distributions of mitotic figures per mm^2^ in the validation datasets are shown in Figs 2 and S3. The number of detected mitotic figures per mm^2^ varied between cancer types, ranging from a median number of mitotic figures per mm^2^ of 2.01 (IQR 1.34–3.15) in prostate cancer to 6.24 (IQR 4.41–9.37) colorectal liver metastases.

In the uterine sarcomas test dataset, an increased number of mitotic figures per mm^2^ detected by the model was a significant marker in both univariable analysis (HR 1.67, *P* < 0.001; Fig. S3) and multivariable analysis (HR 1.63, *P* < 0.001; Fig. S4).

The primary analysis demonstrated that an increased number of detected mitotic figures per mm^2^ was a significant marker of poor outcome in all the validated cancer types except colorectal cancer (Table 3 and Tables S5–S17).

**Table 3:** Primary analysis results. Univariable Cox regression analysis of automatically detected mitotic figures per mm^2^ in the validation datasets. OUS, Oslo University Hospital, SUH, Stavanger University Hospital, VHT, Vestfold Hospital Trust, UNN, University Hospital of North Norway.

Validation dataset	HR (95% CI)	*P*	Endpoint
Prostate cancer OUS 1	1.49 (1.01–2.19)	0.046	Time to recurrence
Prostate cancer OUS 2	2.69 (1.74–4.15)	< 0.001	Biochemical recurrence, PSA ≥ 0.4 ng·mL^−1^
Prostate cancer VHT	1.69 (1.12–2.56)	0.013	Biochemical recurrence, PSA ≥ 0.4 ng·mL^−1^
Prostate cancer UNN	1.22 (0.98–1.51)	0.069	Biochemical recurrence, PSA ≥ 0.4 ng·mL^−1^
Bladder cancer SUH	1.64 (1.09–2.48)	0.018	Time to stage progression
Breast cancer 1990–1998 SUH	1.70 (1.31–2.21)	< 0.001	Time to distant recurrence
Breast cancer 2000–2004 SUH	1.34 (1.05–1.70)	0.018	Time to distant recurrence
Endometrial cancer OUS	1.42 (1.23–1.63)	< 0.001	Time to recurrence
Lung cancer OUS	1.36 (1.18–1.56)	< 0.001	Cancer‐specific survival
Lung cancer UNN	1.19 (1.00–1.42)	0.052	Cancer‐specific survival
Colorectal cancer Cheltenham	1.00 (0.85–1.18)	0.986	Cancer‐specific survival
Colorectal cancer QUASAR2	0.86 (0.69–1.08)	0.195	Cancer‐specific survival
Liver metastases Liverpool	1.28 (1.02–1.59)	0.033	Overall survival

In prostate cancer, an increased number of mitotic figures per mm^2^ was significantly associated with poor outcome in the datasets from Oslo University Hospital and Vestfold Hospital Trust, and close to the significance level in the dataset from the University hospitals of Northern Norway. After exclusion of samples with poor staining quality and patients who did not reach the expected PSA drop after surgery (PSA nadir), the marker was statistically significant also in the latter dataset (Table S8). In bladder cancer, the two breast cancer datasets and endometrial cancer the method was significant. In the lung cancer dataset from Oslo University Hospital, the method was also significant, whereas it was close to the significance level in the dataset from the University hospitals of Northern Norway. In colorectal cancer the number of mitotic figures per mm^2^ was not significant in the primary analyses, whereas the mitotic figure count was a significant marker of overall survival in liver metastases from colorectal cancer.

Secondary analyses of mitotic figures per mm^2^ categorised based on quartiles in the individual validation datasets showed similar trends as the primary analysis (Fig. 3), although statistical significance was more frequent in the primary analyses with mitotic figures per mm^2^ represented as a continuous variable.

In secondary multivariable analyses (Tables S18–S30), the mitotic figure marker was significant in lung cancer and colorectal cancer liver metastases. Secondary analyses with mitotic figures analysed as a continuous variable with other endpoints are shown in Table S32. These analyses were in line with the results using the primary endpoints.

The frequency of mitotic figures per mm^2^ in the most mitotic‐figure‐dense 2 mm^2^ region was compared with the original representation, that is the number of detected mitotic figures per mm^2^ for the entire tumour region. The mitotic figure representations were correlated (Fig. S4). In the breast cancer dataset originating from Stavanger University Hospital 1990–1998, a higher number of mitotic figures per mm^2^ was reported in the most mitotic‐figure‐dense 2 mm^2^ region [median 7.5 (IQR 4.5–12.0)] compared to both automatic counts in the entire tumour region [median 2.8 (IQR 1.7–4.3)] and manual counts in 10 HPF [median 3 (IQR 1–9)]. A similar prognostic impact was observed in univariable survival analysis with the mitotic figure counts as a continuous variable of either automatic representation or manual counts (Table S31 and Fig. S5).

## Discussion

The results demonstrated that automatic mitotic figure counting has the potential to supplement and replace the manual mitosis counting carried out in routine clinical pathology. The automatic measurements of mitotic figures in different cancer types correlates well with proliferations rates presented in the literature, with the lowest mitotic figure frequency observed in prostate cancer and the highest in colorectal cancer and liver metastases from colorectal cancer [17].

The predefined primary analyses (Table 3) showed that automatically detected mitotic figures are prognostic markers in cancer types where mitotic figures already are established markers. Furthermore, the results demonstrated that mitosis counting has the potential as a prognostic marker in cancer types where it is currently not used, such as prostate cancer, bladder cancer and liver metastases. These results were achieved with a method trained on only the TUPAC16 dataset, which is modest in size and only contains breast cancer cases [7]. Other publicly available datasets, such as MIDOG++ [18], could have been included during training. However, the main aim of this study was to show that a deep learning model for mitosis detection trained on a relatively simple dataset could generalise to external cohorts and multiple cancer types. To the best of our knowledge, this is the largest validation of an automated method for counting mitotic figures. Our validation results confirmed that automated mitosis detection can be generalised; however, incorporating additional training data would likely improve both performance and robustness. Recently, Shen *et al*. created the largest available dataset of mitotic figures, demonstrating the benefit of aggregating multi‐centre data. Their study showed that F1‐scores increased in correlation with the number of mitotic figures, ultimately outperforming previous state‐of‐the‐art models in held‐out test sets [19]. The authors attribute these performance gains to both the increased dataset size and their two‐stage OMG‐Net architecture, which utilises the Segment Anything Model [20] as a first stage to generate precise nuclear masks, followed by a second‐stage classifier to distinguish mitotic figures.

A limitation of this study is the lack of an external dataset with expert pathologist annotations for individual mitotic figures. While a direct comparison between model detections and manual annotations would provide deeper insights into generalisability, the annotation process is notoriously time‐consuming and subject to high interobserver variability [21]. We mitigate this issue by validating against clinical endpoints. Future studies are planned to evaluate the model's mitosis detections through both qualitative review and quantitative assessment.

Mitotic figure counting was not a prognostic marker in colorectal cancer in the primary analysis. Normal colorectal tissue is highly proliferative, which may be the explanation why mitosis counting is not a good marker in this cancer type. Performance may also vary across tumour subtypes; for instance, Balkenhol *et al*. [22] previously observed that mitotic counts lack prognostic significance in triple‐negative breast cancer. Shen *et al*. [19] also reported lower performance in neuroendocrine tumours compared to breast carcinoma and melanoma. Further investigation is required to identify tumour types and subtypes where automated algorithms might underperform.

In multivariable analyses, mitotic figures were significant in lung cancer and colorectal cancer liver metastases, but not in the other cancer types. The magnitude of the prognostic impact of mitotic figures combined with the study sizes may explain these results. Despite lack of significance in some multivariable analyses, the prognostic impact of objective and standardised counting of mitotic figures should not be underestimated as a tool in clinical routine pathology. While several assessments in pathology are strong prognostic markers, they are also to a large extent manual and subjective with significant interobserver variability. Automatic mitotic figure counting, on the other hand, is an easily accessible and reproducible marker.

Mitotic figures are normally counted manually in 10 HPFs corresponding to about 1–3 mm^2^, selected as a consecutive region with high cellular proliferation. The automatic mitotic figure counting was performed in the entire tumour region and a direct comparison between manual and automatic mitotic figure counting was therefore not done. However, we mimicked the pathologist approach in *post hoc* analyses by identifying the most mitotic figure‐dense region of size 2 mm^2^ and used that to represent the sample. The results from the hotspot approach and the original approach in uterine sarcomas and breast cancer showed no differences in prognostic impact in univariable analysis with the number of mitotic figures per mm^2^ represented as a continuous variable (Table S31). The observed results indicate that the mitotic figure density is a robust prognostic marker, not sensitive to the tumour region where it is measured. A third representation of mitotic counts is the mitotic index, which is the ratio of mitotic figures to the total number of malignant cells. This approach accounts for variations in tumour cellularity. Ibrahim *et al*. [23] performed a study comparing the three methods and found that only the two methods using mitotic density (tumour area and hotspot) were correlated with the visual scoring and Ki67 scores.

The categorisation of the manual mitotic figure count allows for practical use of the method in clinical practice, for example, as part of the Nottingham grading system in breast cancer [24]. Compared to manual counting in the breast cancer datasets, the automatic method identified more mitotic figure candidates. The restriction of manual counting to include only unambiguous mitotic figures may be part of the explanation. A similar prognostic impact in univariable Cox regression analysis with mitotic figure counts as a continuous variable supports that similar prognostic patterns are described with the two approaches. An adaption is required to integrate automatic counting in established grading systems.

The feature representations mitotic figures per mm^2^ and categorisation with thresholds based on quartiles were chosen for consistency in result presentation and because the mitotic figure density varies between cancer types (Figs 2 and S3), thus making it unlikely that a common categorisation based on absolute threshold values can be prognostically meaningful for all cancer types. The categorisation should instead be identified separately for each cancer type by reflecting the mitotic figure density distribution and provide clinically useful risk stratification of patients.

## Conclusion

It is proven beyond any doubt that mitotic figure counting is a prognostic marker in several cancer types and that mitotic figures can be detected by computers and deep learning. Broad and strong documentation is provided in this study, including that automatic mitotic figure counting should be expanded to cancer types currently not counted in clinical routine. With automatic figure counting and the implementation of digital pathology in hospitals, one can easily offer this service in most, if not all, cancer types. We suggest that this method is included together with other baseline characteristics for all cancer patients.

## Conflict of interest

SDR reports being employed and having stocks in DoMore Diagnostics AS. MXI, MP, AK, OJS, TT, JAN, HAA, WK, MN, KL and TSH report having stocks in DoMore Diagnostics AS. DK reports honoraria from Takeda, Chugai, Lilly, MSD, Ono, Seagen, Guardant Health, Eisai, Taiho, Bristol Myers Squibb, Daiichi‐Sankyo, Pfizer, Merckbiopharma and Sysmex: research funding from Ono, MSD, Novartis, Servier, Janssen, IQVIA, Syneoshealth, CIMIC and Cimicshiftzero. All remaining authors have declared no conflict of interest.

## Author contributions

TSH, HED and AK conceptualised the study. JK and TSH developed the methodology. JK, FJ, AK, SDR, O‐JS, TT and HMM were responsible for the software development. JK, MXI and TSH performed the validation. TSH, KL, AK and JK performed the formal analysis. MXI, KC, WK, ER (Rewcastle), ML, VK, EJ, RJ, OTB, BB, EH, L‐TRB, ER (Richardsen), SA, TD, KL, GK, NAS, MN and DK acquired and provided the validation data. MXI, MP, O‐JS, KC and WK curated the validation data. JK and MXI were responsible for visualisation. HED, TSH, HAA and JAN supervised the project, and HED, TSH and MXI administered the project. HED, TSH and HAA were acquired the funding. TSH, JK, MXI and FJ wrote the original draft. All authors contributed to reviewing and editing of the final manuscript.

## Supporting information


**Table S1.** Clinical applications of mitotic figure counting.
**Table S2.** Datasets and ethical approvals.
**Table S3.** Patient characteristics and univariable analysis, uterine sarcomas test dataset.
**Table S4.** Multivariable analysis in the uterine sarcomas test dataset.
**Table S5.** Patient characteristics and univariable analysis, prostate cancer, Oslo University Hospital 1.
**Table S6.** Patient characteristics and univariable analysis, prostate cancer, Oslo University Hospital 2.
**Table S7.** Patient characteristics and univariable analysis, prostate cancer, Vestfold Hospital Trust.
**Table S8.** Patient characteristics and univariable analysis, prostate cancer, University hospitals of Northern Norway.
**Table S9.** Patient characteristics and univariable analysis, bladder cancer, Stavanger University Hospital.
**Table S10.** Patient characteristics and univariable analysis, breast cancer 1990–1998, Stavanger University Hospital.
**Table S11.** Patient characteristics and univariable analysis, breast cancer 2000–2004, Stavanger University Hospital.
**Table S12.** Patient characteristics and univariable analysis, endometrial cancer, Oslo University Hospital.
**Table S13.** Univariable analysis, lung cancer, Oslo University Hospital.
**Table S14.** Univariable analysis, lung cancer, University hospitals of Northern Norway.
**Table S15.** Patient characteristics and univariable analysis, colorectal cancer, Cheltenham General Hospital.
**Table S16.** Patient characteristics and univariable analysis, colorectal cancer, QUASAR 2.
**Table S17.** Patient characteristics and univariable analysis, colorectal cancer liver metastases, Liverpool.
**Table S18.** Multivariable analysis, prostate cancer, Oslo University Hospital 1.
**Table S19.** Multivariable analysis, prostate cancer, Oslo University Hospital 2.
**Table S20.** Multivariable analysis, prostate cancer, Vestfold Hospital Trust.
**Table S21.** Multivariable analysis, prostate cancer, university hospitals of Northern Norway.
**Table S22.** Multivariable analysis, bladder cancer, Stavanger University Hospital.
**Table S23.** Multivariable analysis, breast cancer 1990–1998, Stavanger University Hospital.
**Table S24.** Multivariable analysis, breast cancer 2000–2004, Stavanger University Hospital.
**Table S25.** Multivariable analysis, endometrial cancer, Oslo University Hospital.
**Table S26.** Multivariable analysis, lung cancer, Oslo University Hospital.
**Table S27.** Multivariable analysis, lung cancer, University hospitals of Northern Norway.
**Table S28.** Multivariable analysis, colorectal cancer, Cheltenham General Hospital.
**Table S29.** Multivariable analysis, colorectal cancer, QUASAR 2.
**Table S30.** Multivariable analysis, colorectal cancer liver metastases, Liverpool.
**Table S31.** Comparison of prognostic impact across different mitotic figure counting methods in uterine sarcomas and breast cancer cohorts.
**Table S32.** Mitotic figure counts analysed with other endpoints.
**Fig. S1.** Performance metrics vs training iteration for the 5 independently trained Mask RCNN models.
**Fig. S2.** Performance metrics in the train and tune datasets vs score threshold for the ensemble model.
**Fig. S3.** Histograms of detected mitotic figures per mm^2^ in the validation datasets.
**Fig. S4.** Comparison of mitotic figure counting in hotspots and the entire tumour region in uterine sarcomas and breast cancer.
**Fig. S5.** Kaplan–Meier survival curves for original and hotspot representation of mitotic figures in uterine sarcomas and breast cancer.
**Appendix S1.** Protocol for the validation of a deep learning system for automatic mitotic figure counting.

## Data Availability

Individual patient level data can be made available to other researchers upon reasonable request by contacting the corresponding author, subject to approval by the relevant people or review board at the institutions that provided the original data.

## References

[1] Boveri T (1914) Zur Frage der Entstehung Maligner Tumoren. Verlag von Gustav Fischer, Jena.

[2] Whitfield ML , George LK , Grant GD and Perou CM (2006) Common markers of proliferation. Nat Rev Cancer 6, 99–106. 16491069 10.1038/nrc1802

[3] Bloom HJ and Richardson WW (1957) Histological grading and prognosis in breast cancer; a study of 1409 cases of which 359 have been followed for 15 years. Br J Cancer 11, 359–377. 13499785 10.1038/bjc.1957.43 PMC2073885

[4] van Diest PJ , Baak JP , Matze‐Cok P , Wisse‐Brekelmans EC , van Galen CM , Kurver PH , Bellot SM , Fijnheer J , van Gorp LH and Kwee WS (1992) Reproducibility of mitosis counting in 2469 breast cancer specimens: results from the multicenter morphometric mammary carcinoma project. Hum Pathol 23, 603–607. 1592381 10.1016/0046-8177(92)90313-r

[5] Gemer O , Uriev L , Voldarsky M , Gdalevich M , Ben‐Dor D , Barak F , Anteby EY and Lavie O (2009) The reproducibility of histological parameters employed in the novel binary grading systems of endometrial cancer. Eur J Surg Oncol 35, 247–251. 18775628 10.1016/j.ejso.2008.07.010

[6] Veta M , van Diest PJ , Willems SM , Wang H , Madabhushi A , Cruz‐Roa A , Gonzalez F , Larsen AB , Vestergaard JS , Dahl AB *et al*. (2015) Assessment of algorithms for mitosis detection in breast cancer histopathology images. Med Image Anal 20, 237–248. 25547073 10.1016/j.media.2014.11.010

[7] Veta M , Heng YJ , Stathonikos N , Bejnordi BE , Beca F , Wollmann T , Rohr K , Shah MA , Wang D , Rousson M *et al*. (2019) Predicting breast tumor proliferation from whole‐slide images: the TUPAC16 challenge. Med Image Anal 54, 111–121. 30861443 10.1016/j.media.2019.02.012

[8] Aubreville M , Stathonikos N , Bertram CA , Klopfleisch R , Ter Hoeve N , Ciompi F , Wilm F , Marzahl C , Donovan TA , Maier A *et al*. (2023) Mitosis domain generalization in histopathology images – the MIDOG challenge. Med Image Anal 84, 102699. 36463832 10.1016/j.media.2022.102699

[9] Tellez D , Balkenhol M , Otte‐Holler I , van de Loo R , Vogels R , Bult P , Wauters C , Vreuls W , Mol S , Karssemeijer N *et al*. (2018) Whole‐slide mitosis detection in H&E breast histology using PHH3 as a reference to train distilled stain‐invariant convolutional networks. IEEE Trans Med Imaging 37, 2126–2136. 29994086 10.1109/TMI.2018.2820199

[10] Nateghi R , Danyali H and Helfroush MS (2021) A deep learning approach for mitosis detection: application in tumor proliferation prediction from whole slide images. Artif Intell Med 114, 102048. 33875159 10.1016/j.artmed.2021.102048

[11] Aubreville M , Stathonikos N , Donovan TA , Klopfleisch R , Ammeling J , Ganz J , Wilm F , Veta M , Jabari S , Eckstein M *et al*. (2024) Domain generalization across tumor types, laboratories, and species – insights from the 2022 edition of the mitosis domain generalization challenge. Med Image Anal 94, 103155. 38537415 10.1016/j.media.2024.103155

[12] Stathonikos N , Aubreville M , de Vries S , Wilm F , Bertram CA , Veta M and van Diest PJ (2024) Breast cancer survival prediction using an automated mitosis detection pipeline. J Pathol Clin Res 10, e70008. 39466133 10.1002/2056-4538.70008 PMC11514500

[13] Abeler VM , Royne O , Thoresen S , Danielsen HE , Nesland JM and Kristensen GB (2009) Uterine sarcomas in Norway. A histopathological and prognostic survey of a total population from 1970 to 2000 including 419 patients. Histopathology 54, 355–364. 19236512 10.1111/j.1365-2559.2009.03231.x

[14] Macenko M , Niethammer M , Marron JS , Borland D , Woosley JT , Xiaojun G , Schmitt C and Thomas NE (2009) A method for normalizing histology slides for quantitative analysis. paper presented at the 2009 IEEE International Symposium on Biomedical Imaging: from nano to macro.

[15] Harrell FE Jr , Califf RM , Pryor DB , Lee KL and Rosati RA (1982) Evaluating the yield of medical tests. JAMA 247, 2543–2546. 7069920

[16] Kleppe A , Skrede OJ , De Raedt S , Liestøl K , Kerr DJ and Danielsen HE (2021) Designing deep learning studies in cancer diagnostics. Nat Rev Cancer 21, 199–211. 33514930 10.1038/s41568-020-00327-9

[17] Bielski CM , Zehir A , Penson AV , Donoghue MTA , Chatila W , Armenia J , Chang MT , Schram AM , Jonsson P , Bandlamudi C *et al*. (2018) Genome doubling shapes the evolution and prognosis of advanced cancers. Nat Genet 50, 1189–1195. 30013179 10.1038/s41588-018-0165-1 PMC6072608

[18] Aubreville M , Wilm F , Stathonikos N , Breininger K , Donovan TA , Jabari S , Veta M , Ganz J , Ammeling J , van Diest PJ *et al*. (2023) A comprehensive multi‐domain dataset for mitotic figure detection. Sci Data 10, 484. 37491536 10.1038/s41597-023-02327-4 PMC10368709

[19] Shen Z , Simard M , Brand D , Andrei V , Al‐Khader A , Oumlil F , Trevers K , Butters T , Haefliger S , Kara E *et al*. (2024) A deep learning framework deploying segment anything to detect pan‐cancer mitotic figures from haematoxylin and eosin‐stained slides. Commun Biol 7, 1674. 39702417 10.1038/s42003-024-07398-6 PMC11659629

[20] Kirillov A , Mintun E , Ravi N , Mao H , Rolland C , Gustafson L , Xiao T , Whitehead S , Berg AC , Lo W‐Y *et al*. (2023) Segment anything. *arXiv preprint arXiv*: 2304.02643.

[21] Veta M , van Diest PJ , Jiwa M , Al‐Janabi S and Pluim JP (2016) Mitosis counting in breast cancer: object‐level Interobserver agreement and comparison to an automatic method. PLoS One 11, e0161286. 27529701 10.1371/journal.pone.0161286 PMC4987048

[22] Balkenhol MCA , Bult P , Tellez D , Vreuls W , Clahsen PC , Ciompi F and van der Laak J (2019) Deep learning and manual assessment show that the absolute mitotic count does not contain prognostic information in triple negative breast cancer. Cell Oncol 42, 555–569. 10.1007/s13402-019-00445-z PMC12994351 30989469

[23] Ibrahim A , Jahanifar M , Wahab N , Toss MS , Makhlouf S , Atallah N , Lashen AG , Katayama A , Graham S , Bilal M *et al*. (2024) Artificial intelligence‐based mitosis scoring in breast cancer: clinical application. Mod Pathol 37, 100416. 38154653 10.1016/j.modpat.2023.100416

[24] Tan PH , Ellis I , Allison K , Brogi E , Fox SB , Lakhani S , Lazar AJ , Morris EA , Sahin A , Salgado R *et al*. (2020) The 2019 World Health Organization classification of tumours of the breast. Histopathology 77, 181–185. 32056259 10.1111/his.14091

[25] Ersvaer E , Kildal W , Vlatkovic L , Cyll K , Pradhan M , Kleppe A , Hveem TS , Askautrud HA , Novelli M , Waehre H *et al*. (2020) Prognostic value of mitotic checkpoint protein BUB3, cyclin B1, and pituitary tumor‐transforming 1 expression in prostate cancer. Mod Pathol 33, 905–915. 31801961 10.1038/s41379-019-0418-2 PMC7190565

[26] Ersvaer E , Hveem TS , Vlatkovic L , Brennhovd B , Kleppe A , Tobin KAR , Pradhan M , Cyll K , Waehre H , Kerr DJ *et al*. (2020) Prognostic value of DNA ploidy and automated assessment of stroma fraction in prostate cancer. Int J Cancer 147, 1228–1234. 31846064 10.1002/ijc.32832

[27] Andersen S , Richardsen E , Nordby Y , Ness N , Størkersen O , Al‐Shibli K , Donnem T , Bertilsson H , Busund LT , Angelsen A *et al*. (2014) Disease‐specific outcomes of radical prostatectomies in northern Norway; a case for the impact of perineural infiltration and postoperative PSA‐doubling time. BMC Urol 14, 49. 24929427 10.1186/1471-2490-14-49 PMC4067377

[28] Hald SM , Rakaee M , Martinez I , Richardsen E , Al‐Saad S , Paulsen EE , Blix ES , Kilvaer T , Andersen S , Busund LT *et al*. (2018) LAG‐3 in non‐small‐cell lung cancer: expression in primary tumors and metastatic lymph nodes is associated with improved survival. Clin Lung Cancer 19, 249–259. 29396238 10.1016/j.cllc.2017.12.001

[29] Rakaee M , Busund LR , Jamaly S , Paulsen EE , Richardsen E , Andersen S , Al‐Saad S , Bremnes RM , Donnem T and Kilvaer TK (2019) Prognostic value of macrophage phenotypes in resectable non‐small cell lung cancer assessed by multiplex immunohistochemistry. Neoplasia 21, 282–293. 30743162 10.1016/j.neo.2019.01.005 PMC6369140

[30] Skaland I , Janssen EA , Gudlaugsson E , Klos J , Kjellevold KH , Søiland H and Baak JP (2009) Validating the prognostic value of proliferation measured by phosphohistone H3 (PPH3) in invasive lymph node‐negative breast cancer patients less than 71 years of age. Breast Cancer Res Treat 114, 39–45. 18373192 10.1007/s10549-008-9980-x

[31] Jonsdottir K , Janssen SR , Da Rosa FC , Gudlaugsson E , Skaland I , Baak JP and Janssen EA (2012) Validation of expression patterns for nine miRNAs in 204 lymph‐node negative breast cancers. PLoS One 7, e48692. 23144930 10.1371/journal.pone.0048692 PMC3492447

[32] Egeland NG , Austdal M , van Diermen‐Hidle B , Rewcastle E , Gudlaugsson EG , Baak JPA , Skaland I , Janssen EAM and Jonsdottir K (2019) Validation study of MARCKSL1 as a prognostic factor in lymph node‐negative breast cancer patients. PLoS One 14, e0212527. 30856208 10.1371/journal.pone.0212527 PMC6411117

[33] Kvikstad V , Mangrud OM , Gudlaugsson E , Dalen I , Espeland H , Baak JPA and Janssen EAM (2019) Prognostic value and reproducibility of different microscopic characteristics in the WHO grading systems for pTa and pT1 urinary bladder urothelial carcinomas. Diagn Pathol 14, 90. 31412916 10.1186/s13000-019-0868-3 PMC6694469

[34] Lillesand M , Kvikstad V , Mangrud OM , Gudlaugsson E , van Diermen‐Hidle B , Skaland I , Baak JPA and Janssen EAM (2020) Mitotic activity index and CD25+ lymphocytes predict risk of stage progression in non‐muscle invasive bladder cancer. PLoS One 15, e0233676. 32484812 10.1371/journal.pone.0233676 PMC7266352

[35] Petersen VC , Baxter KJ , Love SB and Shepherd NA (2002) Identification of objective pathological prognostic determinants and models of prognosis in dukes' B colon cancer. Gut 51, 65–69. 12077094 10.1136/gut.51.1.65 PMC1773289

[36] Mitchard JR , Love SB , Baxter KJ and Shepherd NA (2010) How important is peritoneal involvement in rectal cancer? A prospective study of 331 cases. Histopathology 57, 671–679. 21083598 10.1111/j.1365-2559.2010.03687.x

[37] Quasar Collaborative , Gray R , Barnwell J , McConkey C , Hills RK , Williams NS and Kerr DJ (2007) Adjuvant chemotherapy versus observation in patients with colorectal cancer: a randomised study. Lancet 370, 2020–2029. 18083404 10.1016/S0140-6736(07)61866-2

