# Supplementary material for: Applicability of mitotic figure counting by deep learning: a development and pan‐cancer validation study
Source: FEBS Open Bio. 2026 Feb 12;16(7):1259–70. doi: 10.1002/2211-5463.70210 (PMC13327041; doi:10.1002/2211-5463.70210)
Supplement: Supplementary file 1 — Table S1. Clinical applications of mitotic figure counting. Table S2. Datasets and ethical approvals. Table S3. Patient characteristics and univariable analysis, uterine sarcomas test dataset. Table S4. Multivariable analysis in the uterine sarcomas test dataset. Table S5. Patient characteristics and univariable analysis, prostate cancer, Oslo University Hospital 1. Table S6. Patient characteristics and univariable analysis, prostate cancer, Oslo University Hospital 2. Table S7. Patient characteristics and univariable analysis, prostate cancer, Vestfold Hospital Trust. Table S8. Patient characteristics and univariable analysis, prostate cancer, University hospitals of Northern Norway. Table S9. Patient characteristics and univariable analysis, bladder cancer, Stavanger University Hospital. Table S10. Patient characteristics and univariable analysis, breast cancer 1990–1998, Stavanger University Hospital. Table S11. Patient characteristics and univariable analysis, breast cancer 2000–2004, Stavanger University Hospital. Table S12. Patient characteristics and univariable analysis, endometrial cancer, Oslo University Hospital. Table S13. Univariable analysis, lung cancer, Oslo University Hospital. Table S14. Univariable analysis, lung cancer, University hospitals of Northern Norway. Table S15. Patient characteristics and univariable analysis, colorectal cancer, Cheltenham General Hospital. Table S16. Patient characteristics and univariable analysis, colorectal cancer, QUASAR 2. Table S17. Patient characteristics and univariable analysis, colorectal cancer liver metastases, Liverpool. Table S18. Multivariable analysis, prostate cancer, Oslo University Hospital 1. Table S19. Multivariable analysis, prostate cancer, Oslo University Hospital 2. Table S20. Multivariable analysis, prostate cancer, Vestfold Hospital Trust. Table S21. Multivariable analysis, prostate cancer, university hospitals of Northern Norway. Table S22. Multivariable analysis, bladder cancer, Stavanger U [file FEB4-16-1259-s001.pdf]

Supplementary information

Table of contents

Supporting

Supporting Table 1: Clinical applications of mitotic figure counting....................................3
Supporting Table 2: Datasets and ethical approvals...4
Supporting Table 3: Patient characteristics and univariable analysis, uterine sarcomas test dataset 5
Supporting Table 4: Multivariable analysis in the uterine sarcomas test dataset 6
Supporting Table 5: Patient characteristics and univariable analysis, prostate cancer, Oslo University Hospital 1 7
Supporting Table 6: Patient characteristics and univariable analysis, prostate cancer, Oslo University Hospital 2 8
Supporting Table 7: Patient characteristics and univariable analysis, prostate cancer, Vestfold Hospital Trust...9
Supporting Table 8: Patient characteristics and univariable analysis, prostate cancer, University hospitals of Northern Norway 10
Supporting Table 9: Patient characteristics and univariable analysis, bladder cancer, Stavanger University Hospital 11
Supporting Table 10: Patient characteristics and univariable analysis, breast cancer 1990-1998, Stavanger University Hospital 12
Supporting Table 11: Patient characteristics and univariable analysis, breast cancer 2000-2004, Stavanger University Hospital 13
Supporting Table 12: Patient characteristics and univariable analysis, endometrial cancer, Oslo University Hospital...14
Supporting Table 13: Univariable analysis, lung cancer, Oslo University Hospital 15
Supporting Table 14: Univariable analysis, lung cancer, University hospitals of Northern Norway...16
Supporting Table 15: Patient characteristics and univariable analysis, colorectal cancer, Cheltenham General Hospital 17
Supporting Table 16: Patient characteristics and univariable analysis, colorectal cancer, QUASAR 2 18
Supporting Table 17: Patient characteristics and univariable analysis, colorectal cancer liver metastases, Liverpool...19
Supporting Table 18: Multivariable analysis, prostate cancer, Oslo University Hospital 1..20
Supporting Table 19: Multivariable analysis, prostate cancer, Oslo University Hospital 2..21
Supporting Table 20: Multivariable analysis, prostate cancer, Vestfold Hospital Trust.......22
Supporting Table 21: Multivariable analysis, prostate cancer, university hospitals of Northern Norway 23
Supporting Table 22: Multivariable analysis, bladder cancer, Stavanger University Hospital 24
Supporting Table 23: Multivariable analysis, breast cancer 1990-1998, Stavanger University Hospital..25
Supporting Table 24: Multivariable analysis, breast cancer 2000-2004, Stavanger University Hospital..26
Supporting Table 25: Multivariable analysis, endometrial cancer, Oslo University Hospital 27
Supporting Table 26: Multivariable analysis, lung cancer, Oslo University Hospital 28

Supporting Table 27: Multivariable analysis, lung cancer, University hospitals of Northern Norway 29
Supporting Table 28:Multivariable analysis, colorectal cancer, Cheltenham General Hospital 30
Supporting Table 29: Multivariable analysis, colorectal cancer, QUASAR 2 31
Supporting Table 30: Multivariable analysis, colorectal cancer liver metastases, Liverpool32
Supporting Table 31: Prognostic impact of automatic mitotic figure counting in the entire tumour region, in the most mitotic figure dense 2 mm^2 hotspot, and manually counted mitotic figures in the uterine sarcomas testset and the two breast cancer datasets from Stavanger University Hospital. 33
Supporting Table 32: Mitotic figure counts analysed with other endpoints 34

Supporting Figure 1: Performance metrics vs training iteration for the 5 independently trained Mask RCNN models. The iteration with the highest f1-score was selected and is indicated with a circle in the plots. 36
Supporting Figure 2: Performance metrics in the train and tune datasets vs score threshold for the ensemble model. A score threshold of 0.4 was selected. Detections with ensemble score below 0.4 were excluded. 37
Supporting Figure 3: Histograms of detected mitotic figures per mm2 in the validation datasets 39
Supporting Figure 4: Detected mitotic figures per mm^2 in the most mitotic figure dense 2 mm^2 circle shaped hotspots vs in the entire tumour region in uterine sarcomas test dataset (left) and the two breast cancer datasets (right). 40
Supporting Figure 5: Kaplan-Meier survival curves for original and hotspot representation for mitotic figures in the uterine sarcomas testset and the two breast cancer datasets from Stavanger University Hospital. 41

The protocol........ pages 42-71

Cancer	How used	Guideline
Breast cancer	Mitotic figure counting part of grading	ESMO (does not specify grading type). NCCN does not include mitotic figure counting (has Ki-67).
Cervical cancer	Abundant mitotic activity used as histologic description for neuroendocrine carcinoma of the cervix (NECC)	NCCN
Gastrointestinal stromal tumours	To assess malignant potential (together with other markers)	NCCN
Chronic lymphocytic leukemia (CLL)/small lymphocytic lymphoma	>2·4 mitotic figures per proliferation center used in combination with other markers to diagnose "CLL with expanded proliferation centers" or "accelerated CLL"	NCCN
Non-small cell lung carcinomas	Distinguish typical carcinoids from atypical carcinoids	NCCN
Ovarian cancer	Mitotic figure counts is part of the diagnosis of low-grade and high-grade serous carcinoma.	NCCN, ESMO
Non-epithelial ovarian cancer	"Advanced-stage and recurrent SCSTs: management ": "Patients with steroid cell tumours that are pleomorphic, large, at an advanced stage or with an increased mitotic count should be treated with additional postoperative platinum-based ChT, either BEP (if not previously used) or a taxane–platinum combination"	ESMO
Burkitt lymphoma	Characteristics of burkitt lymphoma: "Mitoses and apoptotic bodies are often numerous."	NCCN
Dermatofibrosarcoma protuberans	Fibrosarcomatous transformation (FS-DFSP) is characterized by transition from storiform to a herringbone pattern, with a higher degree of cellularity, cytologic atypia, mitotic activity (>5/10 HPF), and frequent loss of CD34 immunostaining.	NCCN
Gestational trophoblastic neoplasia	Mitotic figure counts (>5/10 HPFs) used together with other markers to identify candidate patients for systemic therapy after surgery.	NCCN
Cutaneous melanoma	Mitoses per mm^2 is an independent predictor of patient outcome and should be reported for all lesions (both guidelines). Used in the separation of stage IA and IB (not in AJCC Cancer Staging Manual Eight Edition (2017)) (NCCN).	NCCN, ESMO
Neuroendocrine and adrenal tumours	Mitotic figure counting used in histologic classification.	NCCN
Small cell lung cancer	Careful counting of mitoses is essential, because it is the most important histologic criterion for distinguishing SCLC from typical and atypical Carcinoids.	NCCN
Soft tissue sarcoma	Mitotic figure counting used in the assessment of histologic grade	NCCN
Thymomas and thymic carcinomas	Mitotic figure counting used in the assessment of thymoma subtype (atypical type A variant have increased mitotic count).	NCCN
Uterine sarcomas	Mitotic figure counting used in the assessment of histologic type and malignancy potential	NCCN

Supporting Table 1: *Clinical applications of mitotic figure counting*

Dataset	Regional ethics committee approval
Prostate cancer, Oslo University Hospital 1	S-07443a 2013/476
Prostate cancer, Oslo University Hospital 2	2013/2111
Prostate cancer, Vestfold Hospital Trust	2012/1679
Prostate cancer, University hospitals of Northern Norway	2009/1393
Bladder cancer, Stavanger University Hospital	172005
Breast cancer, Stavanger University Hospital	2010/1241
Endometrial cancer, Oslo University Hospital	2014/701
Lung cancer, Oslo University Hospital	2009/1904
Lung cancer, University hospitals of Northern Norway	2011/2503
Colorectal cancer, Cheltenham General Hospital	2015/1606
Colorectal cancer, QUASAR2	2015/1607
Uterine sarcomas Oslo University Hospital	S-04298, anonymised

***Supporting Table 2**: Datasets and ethical approvals*

Feature	Median (IQR) / Count (%)	HR (95% CI)	P-value Wald	P-value log-rank
Age	56·0 (IQR 48·0 - 65·0)	1·03 (1·02-1·04)	<0·001	
Mitotic figures per mm^2 (log_2-transformed in analysis)	3·4 (IQR 1·4 - 7·3)	1·67 (1·49-1·87)	<0·001	
Histological subtype				<0·001
Leiomyosarcoma	230 (62%)	Ref.		
Endometrial stromal sarcoma	82 (22%)	0·37 (0·26-0·52)	<0·001	
Adenosarcoma	22 (6%)	0·59 (0·33-1·06)	0·076	
Undifferentiated uterine sarcoma	19 (5%)	1·01 (0·60-1·71)	0·97	
Rare tumours inclusing sarcomas NOS	19 (5%)	1·73 (1·05-2·85)	0·031	
Tumour extent				<0·001
Confined to uterus	281 (76%)	Ref.		
Spread outside uterus	91 (24%)	1·96 (1·50-2·56)	<0·001	
Tumour size				<0·001
≤10 cm	274 (74%)	Ref.		
>10 cm	78 (21%)	2·19 (1·65-2·91)	<0·001	
Missing	20 (5%)			
Tumour margins				0·49
Pushing	80 (22%)	Ref.		
Infiltrating	276 (74%)	1·11 (0·83-1·48)	0·49	
Missing	16 (4%)			
Cellular atypia				<0·001
Mild	110 (30%)	Ref.		
Moderate	141 (38%)	3·28 (2·34-4·58)	<0·001	
Severe	116 (31%)	2·75 (1·95-3·89)	<0·001	
Missing	5 (1%)			
Tumour necrosis				<0·001
Absent	93 (25%)	Ref.		
Present	275 (74%)	2·43 (1·76-3·36)	<0·001	
Missing	4 (1%)			
Hyaline necrosis				0·005
Absent	186 (50%)	Ref.		
Present	173 (47%)	1·42 (1·11-1·82)	0·005	
Missing	13 (3%)			
Vascular invasion				0·62
Absent	196 (53%)	Ref.		
Present	155 (42%)	1·07 (0·83-1·37)	0·62	
Missing	21 (6%)			

***Supporting Table 3**: Patient characteristics and univariable analysis, uterine sarcomas test dataset*

**Feature**	**HR (95% CI)**	**P-value feature level**	**P-value Anova**
Mitotic figures per mm^2 (log_2-transformed)	1·63 (1·41-1·90)	<0·001	<0·001
Histological subtype			0·025
Leiomyosarcoma	Ref.		
Endometrial stromal sarcoma	0·53 (0·31-0·88)	0·015	
Adenosarcoma	1·23 (0·63-2·43)	0·54	
Undifferentiated uterine sarcoma	0·81 (0·43-1·54)	0·52	
Rare tumours inclusing sarcomas NOS	1·69 (0·97-2·95)	0·066	
Tumour extent			<0·001
Confined to uterus	Ref.		
Spread outside uterus	2·25 (1·60-3·16)	<0·001	
Tumour size			0·003
≤10 cm	Ref.		
>10 cm	1·66 (1·19-2·31)	0·003	
Tumour margins			0·31
Pushing	Ref.		
Infiltrating	1·18 (0·85-1·65)	0·31	
Cellular atypia			0·006
Mild	Ref.		
Moderate	1·57 (1·03-2·40)	0·038	
Severe	0·99 (0·62-1·58)	0·97	
Tumour necrosis			0·14
Absent	Ref.		
Present	1·35 (0·91-2·00)	0·14	
Hyaline necrosis			0·44
Absent	Ref.		
Present	1·12 (0·84-1·49)	0·44	
Vascular invasion			0·84
Absent	Ref.		
Present	0·97 (0·72-1·31)	0·84	

Supporting Table 4: *Multivariable analysis in the uterine sarcomas test dataset*

Feature	Median (IQR) / Count (%)	HR (95% CI)	P-value Wald	P-value log-rank
Age	62·5 (IQR 58·1 - 66·9)	1·01 (0·97-1·04)	0·64	
Mitotic figures per mm^2 (log$_2$-transformed in analysis)	2·8 (IQR 2·0 - 3·8)	1·49 (1·01-2·19)	0·046	
Preoperative PSA (log$_2$-transformed in analysis)	13·0 (IQR 7·0 - 20·0)	1·74 (1·41-2·16)	<0·001	
Gleason grade group				<0·001
GG1	11 (4%)	NA	1·00	
GG2	92 (36%)	Ref.		
GG3	77 (30%)	4·20 (1·99-8·87)	<0·001	
GG4	44 (17%)	9·70 (4·57-20·57)	<0·001	
GG5	29 (11%)	10·39 (4·73-22·85)	<0·001	
Extraprostatic extension				<0·001
No	55 (22%)	Ref.		
Yes	196 (77%)	5·04 (2·04-12·43)	<0·001	
Missing	2 (1%)			
Seminal vesicle invasion				<0·001
No	187 (74%)	Ref.		
Yes	66 (26%)	4·09 (2·68-6·25)	<0·001	
Surgical margins				0·002
Free	91 (36%)	Ref.		
Not free	162 (64%)	2·18 (1·31-3·64)	0·003	
Lymph node metastasis				0·002
No	239 (94%)	Ref.		
Yes	14 (6%)	2·83 (1·42-5·65)	0·003	

***Supporting Table 5**: Patient characteristics and univariable analysis, prostate cancer, Oslo University Hospital 1*

Feature	Median (IQR) / Count (%)	HR (95% CI)	P-value Wald	P-value log-rank
Age	62·3 (IQR 58·9 - 66·1)	1·04 (1·00-1·09)	0·048	
Mitotic figures per mm^2 (log_2-transformed in analysis)	1·3 (IQR 1·0 - 1·7)	2·69 (1·74-4·15)	<0·001	
Preoperative PSA (log_2-transformed in analysis)	8·2 (IQR 6·5 - 11·4)	2·39 (1·74-3·28)	<0·001	
Gleason grade group				<0·001
GG1	3 (1%)	NA	1·00	
GG2	153 (59%)	Ref.		
GG3	54 (21%)	1·66 (0·85-3·22)	0·14	
GG4	12 (5%)	1·70 (0·51-5·67)	0·39	
GG5	37 (14%)	10·94 (6·31-18·95)	<0·001	
Extraprostatic extension				<0·001
No	166 (64%)	Ref.		
Yes	89 (34%)	3·97 (2·45-6·44)	<0·001	
Missing	4 (2%)			
Seminal vesicle invasion				<0·001
No	228 (88%)	Ref.		
Yes	30 (12%)	5·39 (3·22-9·02)	<0·001	
Missing	1 (0%)			
Surgical margins				<0·001
Free	165 (64%)	Ref.		
Not free	92 (36%)	2·90 (1·80-4·67)	<0·001	
Missing	2 (1%)			
Lymph node metastasis				<0·001
No	252 (97%)	Ref.		
Yes	7 (3%)	6·25 (2·67-14·60)	<0·001	

Supporting Table 6*: Patient characteristics and univariable analysis, prostate cancer, Oslo University Hospital 2*

Feature	Median (IQR) / Count (%)	HR (95% CI)	P-value Wald	P-value log-rank
Age	64·4 (IQR 60·6 - 67·9)	1·02 (0·98-1·06)	0·33	
Mitotic figures per mm^2 (log_2-transformed in analysis)	1·8 (IQR 1·4 - 2·6)	1·69 (1·12-2·56)	0·013	
Preoperative PSA (log_2-transformed in analysis)	8·9 (IQR 6·6 - 13·0)	1·62 (1·23-2·14)	<0·001	
Gleason grade group				<0·001
GG1	33 (10%)	0·79 (0·31-2·03)	0·62	
GG2	168 (52%)	Ref.		
GG3	101 (31%)	3·09 (1·95-4·90)	<0·001	
GG4	20 (6%)	4·67 (2·33-9·36)	<0·001	
GG5	4 (1%)	3·88 (0·92-16·30)	0·064	
Extraprostatic extension				<0·001
No	187 (57%)	Ref.		
Yes	125 (38%)	2·66 (1·73-4·09)	<0·001	
Missing	14 (4%)			
Seminal vesicle invasion				<0·001
No	288 (88%)	Ref.		
Yes	36 (11%)	3·78 (2·33-6·11)	<0·001	
Missing	2 (1%)			
Surgical margins				<0·001
Free	165 (51%)	Ref.		
Not free	161 (49%)	2·47 (1·60-3·82)	<0·001	
Lymph node metastasis				<0·001
No	318 (98%)	Ref.		
Yes	8 (2%)	5·55 (2·56-12·04)	<0·001	

***Supporting Table** 7: Patient characteristics and univariable analysis, prostate cancer, Vestfold Hospital Trust*

Feature	Median (IQR) / Count (%)	HR (95% CI)	P-value Wald	P-value log-rank
Age	62·0 (IQR 58·0 - 66·1)	1·02 (0·99-1·06)	0·25	
Mitotic figures per mm^2 (log_2-transformed in analysis)	2·2 (IQR 1·5 - 3·6)	1·29 (1·01-1·64)	0·043	
Preoperative PSA (log2-transformed in analysis)	8·6 (IQR 6·2 - 12·1)	1·71 (1·38-2·12)	<0·001	
Gleason grade group				<0·001
GG1	103 (25%)	0·41 (0·21-0·79)	0·008	
GG2	195 (48%)	Ref.		
GG3	56 (14%)	2·20 (1·36-3·55)	0·001	
GG4	21 (5%)	2·39 (1·24-4·59)	0·009	
GG5	29 (7%)	4·33 (2·57-7·29)	<0·001	
Missing	2 (0%)			
Pathological T-stage				<0·001
pT2	291 (72%)	Ref.		
pT3	115 (28%)	2·25 (1·56-3·24)	<0·001	
Surgical margins				0·057
Free	200 (49%)	Ref.		
Not free	206 (51%)	1·43 (0·99-2·07)	0·058	
Lymph node invasion				<0·001
No	404 (100%)	Ref.		
Yes	2 (0%)	13·13 (3·18-54·21)	<0·001	

***Supporting Table 8**: Patient characteristics and univariable analysis, prostate cancer, University hospitals of Northern Norway*

Feature	Median (IQR) / Count (%)	HR (95% CI)	P-value Wald	P-value log-rank
Age	72·5 (IQR 62·2 - 80·0)	1·05 (1·01-1·10)	0·009	
Mitotic figures per mm^2 (log_2-transformed in analysis)	3·1 (IQR 1·8 - 6·1)	1·64 (1·09-2·48)	0·018	
Sex				0·13
Female	84 (25%)	Ref.		
Male	246 (75%)	2·46 (0·73-8·24)	0·14	
WHO grade (2004)				<0·001
Low grade	186 (56%)	Ref.		
High grade	144 (44%)	5·39 (2·01-14·46)	<0·001	
Multifocal				0·007
No	196 (59%)	Ref.		
Yes	107 (32%)	3·13 (1·30-7·55)	0·011	
Missing	27 (8%)			
Urothelial carcinoma in situ				<0·001
No	295 (89%)	Ref.		
Yes	35 (11%)	4·01 (1·66-9·68)	0·002	

Supporting Table 9*: Patient characteristics and univariable analysis, bladder cancer, Stavanger University Hospital*

Feature	Median (IQR) / Count (%)	HR (95% CI)	P-value Wald	P-value log-rank
Age	56·0 (IQR 50·0 - 64·0)	0·96 (0·93-0·98)	0·002	
Mitotic figures per mm^2	2·8 (IQR 1·7 - 4·3)	1·70 (1·31-2·21)	<0·001	
Oestrogen receptor status				0·052
Positive	256 (83%)	Ref.		
Borderline	11 (4%)	1·04 (0·25-4·33)	0·95	
Negative	39 (13%)	2·14 (1·14-4·00)	0·018	
Missing	1 (0%)			
Progesterone receptor status				0·068
Positive	194 (63%)	Ref.		
Borderline	54 (18%)	1·66 (0·90-3·07)	0·10	
Negative	58 (19%)	0·59 (0·25-1·42)	0·24	
Missing	1 (0%)			
HER2 status				0·23
Positive	255 (83%)	Ref.		
Borderline	11 (4%)	1·78 (0·55-5·74)	0·34	
Negative	32 (10%)	1·73 (0·84-3·57)	0·14	
Missing	9 (3%)			
Nottingham grade				<0·001
3-5	107 (35%)	Ref.		
6-7	133 (43%)	4·44 (1·71-11·51)	0·002	
8-9	64 (21%)	7·24 (2·73-19·23)	<0·001	
Missing	3 (1%)			
Nuclear atypia				0·002
Mild variation	45 (15%)	NA	1·00	
Moderate variation	161 (52%)	Ref.		
Marked variation	98 (32%)	1·66 (0·97-2·83)	0·063	
Missing	3 (1%)			
Tumour diameter				0·014
≤ 2 cm	254 (83%)	Ref.		
> 2 cm	52 (17%)	2·09 (1·15-3·79)	0·016	
Missing	1 (0%)			

Supporting Table 10: *Patient characteristics and univariable analysis, breast cancer 1990-1998, Stavanger University Hospital*

Feature	Median (IQR) / Count (%)	HR (95% CI)	P-value Wald	P-value log-rank
Age	55·0 (IQR 47·0 - 63·0)	1·03 (1·01-1·05)	0·007	
Mitotic figures per mm^2	3·5 (IQR 2·2 - 7·6)	1·34 (1·05-1·70)	0·018	
Oestrogen receptor status				<0·001
Positive	140 (51%)	Ref.		
Negative	131 (48%)	4·32 (2·33-8·02)	<0·001	
Missing	3 (1%)			
Progesterone receptor status				<0·001
Positive	94 (34%)	Ref.		
Borderline	2 (1%)	7·55 (0·95-59·78)	0·056	
Negative	147 (54%)	3·71 (1·81-7·58)	<0·001	
Missing	31 (11%)			
Nottingham grade				<0·001
3-5	38 (14%)	Ref.		
6-7	105 (38%)	0·59 (0·21-1·62)	0·30	
8-9	122 (45%)	2·43 (1·03-5·75)	0·042	
Missing	9 (3%)			
Nuclear atypia				0·018
Mild variation	13 (5%)	NA	1·00	
Moderate variation	64 (23%)	Ref.		
Marked variation	88 (32%)	2·13 (1·03-4·42)	0·042	
Missing	109 (40%)			
Tumour diameter				<0·001
≤ 2 cm	174 (64%)	Ref.		
> 2 cm	92 (34%)	3·90 (2·24-6·79)	<0·001	
Missing	8 (3%)			
Lymph node status				<0·001
Negative	202 (74%)	Ref.		
Positive	48 (18%)	4·82 (2·71-8·57)	<0·001	
Missing	24 (9%)			

Supporting Table 11*: Patient characteristics and univariable analysis, breast cancer 2000-2004, Stavanger University Hospital*

Feature	Median (IQR) / Count (%)	HR (95% CI)	P-value Wald	P-value log-rank
Age				<0·001
<60	249 (22%)	Ref.		
≥60	883 (78%)	3·01 (2·01-4·52)	<0·001	
Mitotic figures per mm^2 (log_2-transformed in analysis)	2·9 (IQR 1·8 - 4·7)	1·42 (1·23-1·63)	<0·001	
FIGO stage				<0·001
IA	541 (48%)	Ref.		
IB	287 (25%)	1·45 (1·03-2·06)	0·034	
II	78 (7%)	3·85 (2·53-5·85)	<0·001	
IIIA	41 (4%)	2·70 (1·50-4·87)	<0·001	
IIIB	9 (1%)	6·99 (2·82-17·30)	<0·001	
III	176 (16%)	4·42 (3·24-6·05)	<0·001	
Histological type and differenatiation				<0·001
Endometrioid and grade 1	410 (36%)	Ref.		
Endometrioid and grade 2	241 (21%)	1·68 (1·15-2·44)	0·007	
Endometrioid and grade 3	132 (12%)	1·88 (1·22-2·91)	0·004	
Mixed without c/s and grade 1	30 (3%)	0·99 (0·36-2·73)	0·98	
Mixed without c/s and grade 2	13 (1%)	2·96 (1·07-8·16)	0·036	
Mixed without c/s and grade 3	5 (0%)	4·87 (1·52-15·55)	0·008	
Mucinous carcinoma	6 (1%)	1·14 (0·16-8·24)	0·90	
Clear cell adenocarcinoma	28 (2%)	1·72 (0·74-4·00)	0·20	
Serous carcinoma	112 (10%)	3·87 (2·64-5·67)	<0·001	
Mixed with clear cell or serous carcinoma	60 (5%)	2·19 (1·26-3·81)	0·006	
Undifferentiated carcinoma	7 (1%)	2·60 (0·63-10·65)	0·18	
Unclassifiable carcinoma	14 (1%)	1·80 (0·56-5·74)	0·32	
Neuroendocrine tumour	10 (1%)	2·78 (0·87-8·87)	0·085	
Carsinosarcoma	64 (6%)	4·73 (3·05-7·33)	<0·001	
Lymphovascular space invasion				<0·001
No	803 (71%)	Ref.		
Yes	329 (29%)	2·62 (2·06-3·34)	<0·001	
Adjuvant treatment				<0·001
No	750 (66%)	Ref.		
Yes	382 (34%)	1·89 (1·48-2·40)	<0·001	

***Supporting Table 12**: Patient characteristics and univariable analysis, endometrial cancer, Oslo University Hospital*

Feature	Group	HR (95% CI)	p
Mitotic count (average)	log_2(1+mitosis/mm^2) increment	1·36 (1·18-1·56)	<0·0001
Age	10-year increment	1·12 (0·98-1·28)	0·091
Sex	Male vs· Female	1·12 (0·89-1·41)	0·35
Smoking status	Former or current vs· Never	1·45 (0·92-2·28)	0·11
Pathological stage			<0·0001
	I	Ref.	
	II	1·82 (1·40-2·38)	
	III	2·62 (1·92-3·58)	
	IV	6·19 (3·02-12·68)	
ECOG performance status	≥2 vs· <2	1·88 (1·08-3·30)	0·024
Histological type			<0·0001
	Adenocarcinoma	Ref.	
	Squamous cell carcinoma	0·88 (0·68-1·15)	
	Large cell and undifferentiated carcinoma	1·70 (1·00-2·88)	
	Carcinoid tumour	0·06 (0·01-0·40)	
	Other	1·70 (0·96-2·98)	

Supporting Table 13*: Univariable analysis, lung cancer, Oslo University Hospital*

Feature	Group	HR (95% CI)	p
Mitotic count	$\log_2(1+\text{mitosis/mm}^2)$ increment	1·19 (1·00-1·42)	0·052
Age	10-year increment	1·02 (0·88-1·19)	0·77
Sex	Male vs· Female	1·35 (1·00-1·83)	0·051
Smoking status	Former or current vs· Never	0·65 (0·35-1·20)	0·16
Pathological stage			<0·0001
	I	Ref.	
	II	1·64 (1·15-2·32)	
	III	4·19 (2·99-5·86)	
ECOG performance status	≥2 vs· <2	1·14 (0·62-2·09)	0·68
Histological type			0·067
	Adenocarcinoma	Ref.	
	Squamous cell carcinoma	0·77 (0·59-1·02)	
	Large cell and undifferentiated carcinoma	0·00 (0·00-∞)	
	Other	2·42 (0·60-9·85)	

Supporting Table 14*: Univariable analysis, lung cancer, University hospitals of Northern Norway*

Feature	Median (IQR) / Count (%)	HR (95% CI)	P-value Wald	P-value log-rank
Age	71·0 (IQR 64·0 - 78·0)	1·01 (0·99-1·02)	0·36	
Mitotic figures per mm^2 (log_2-transformed in analysis)	4·8 (IQR 3·0 - 7·0)	1·00 (0·85-1·18)	0·99	
Pathological T stage				<0·001
pT1	15 (2%)	Ref.		
pT2	70 (7%)	NA	0·99	
pT3	434 (44%)	2·18 (0·30-15·75)	0·44	
pT4	458 (47%)	8·45 (1·18-60·32)	0·033	
Missing	1 (0%)			
Pathological N stage				<0·001
pN0	450 (46%)	Ref.		
pN1	282 (29%)	2·57 (1·81-3·64)	<0·001	
pN2	246 (25%)	7·19 (5·22-9·90)	<0·001	
Tumour location				0·086
Proximal	349 (36%)	Ref.		
Distal	304 (31%)	0·82 (0·61-1·10)	0·18	
Rectum	325 (33%)	0·72 (0·54-0·97)	0·032	

Supporting Table 15*: Patient characteristics and univariable analysis, colorectal cancer, Cheltenham General Hospital*

Feature	Median (IQR) / Count (%)	HR (95% CI)	P-value Wald	P-value log-rank
Age	65·0 (IQR 59·0 - 71·0)	1·01 (0·99-1·03)	0·18	
Mitotic figures per mm^2 (log$_2$-transformed in analysis)	6·3 (IQR 4·3 - 9·0)	0·86 (0·69-1·08)	0·20	
Pathological T stage				<0·001
pT1	18 (2%)	Ref.		
pT2	74 (7%)	2·32 (0·29-18·29)	0·43	
pT3	584 (52%)	1·82 (0·25-13·11)	0·55	
pT4	397 (35%)	4·07 (0·57-29·25)	0·16	
Missing	51 (5%)			
Pathological N stage				<0·001
pN0	404 (36%)	Ref.		
pN1	509 (45%)	1·55 (1·00-2·41)	0·052	
pN2	185 (16%)	6·45 (4·19-9·92)	<0·001	
Missing	26 (2%)			
Tumour location				0·30
Proximal	456 (41%)	Ref.		
Distal	455 (40%)	0·80 (0·57-1·13)	0·21	
Rectum	165 (15%)	0·72 (0·43-1·20)	0·21	
Missing	48 (4%)			

***Supporting Table 16**: Patient characteristics and univariable analysis, colorectal cancer, QUASAR 2*

Feature	Median (IQR) / Count (%)	HR (95% CI)	P-value Wald	P-value log-rank
Age	65·5 (IQR 60·3 - 73·8)	1·01 (1·00-1·03)	0·12	
Mitotic figures per mm^2 (log_2-transformed in analysis)	6·2 (IQR 4·4 - 9·4)	1·28 (1·02-1·59)	0·033	
Gender				0·15
Female	77 (33%)	Ref.		
Male	156 (67%)	1·28 (0·92-1·77)	0·15	
Synchronous/metachronous				0·51
Synchronous	64 (27%)	Ref.		
Metachronous	169 (73%)	0·89 (0·63-1·25)	0·51	
Size of largest lesion				0·014
≤50mm	177 (76%)	Ref.		
>50mm	56 (24%)	1·54 (1·09-2·18)	0·015	
Number of metastases				0·12
1	106 (45%)	Ref.		
>1	127 (55%)	1·27 (0·93-1·73)	0·13	
Pathological T stage of primary tumour				0·20
pT1	6 (3%)	Ref.		
pT2	36 (15%)	0·47 (0·18-1·25)	0·13	
pT3	148 (64%)	0·51 (0·21-1·27)	0·15	
pT4	42 (18%)	0·69 (0·27-1·78)	0·45	
Missing	1 (0%)			
Pathological N stage of primary tumour				0·036
pN0	90 (39%)	Ref.		
pN1	76 (33%)	1·43 (0·98-2·06)	0·061	
pN2	66 (28%)	1·60 (1·10-2·34)	0·015	
Missing	1 (0%)			
Location of primary tumour				0·21
Right	52 (22%)	Ref.		
Left	180 (77%)	0·79 (0·55-1·14)	0·21	
Missing	1 (0%)			
Extra hepatic disease				0·041
No	208 (89%)	Ref.		
Yes	25 (11%)	1·62 (1·01-2·60)	0·043	
Neoadjuvant therapy				0·081
No	120 (52%)	Ref.		
Yes	113 (48%)	1·31 (0·97-1·78)	0·082	
Adjuvant therapy				0·38
No	101 (43%)	Ref.		
Yes	62 (27%)	1·18 (0·81-1·72)	0·38	
Missing	70 (30%)			

Supporting Table 17*: Patient characteristics and univariable analysis, colorectal cancer liver metastases, Liverpool*

Feature	HR (95% CI)	P-value feature level	P-value Anova
Mitotic figure per mm^2 (log_2-transformed)	1·18 (0·78-1·77)	0·43	0·43
Age	0·97 (0·94-1·01)	0·13	0·13
Preoperative PSA (log_2-transformed)	1·20 (0·96-1·50)	0·11	0·11
Gleason grade group			<0·001
GG1	NA	0·99	
GG2	Ref.		
GG3	2·19 (0·94-5·11)	0·071	
GG4	5·14 (2·14-12·35)	<0·001	
GG5	5·27 (2·12-13·08)	<0·001	
Extraprostatic extension			0·63
No	Ref.		
Yes	1·28 (0·47-3·45)	0·63	
Seminal vesicle invasion			0·003
No	Ref.		
Yes	2·10 (1·30-3·40)	0·003	
Surgical margins			0·34
Free	Ref.		
Not free	1·32 (0·75-2·32)	0·34	
Lymph node metastasis			0·70
No	Ref.		
Yes	1·16 (0·54-2·47)	0·70	

***Supporting Table 18**: Multivariable analysis, prostate cancer, Oslo University Hospital 1*

Feature	HR (95% CI)	P-value feature level	P-value Anova
Mitotic figures per mm^2 (log_2-transformed)	1·43 (0·82-2·49)	0·21	0·21
Age	0·99 (0·94-1·03)	0·54	0·54
Preoperative PSA (log_2-transformed)	2·33 (1·49-3·63)	<0·001	<0·001
Gleason grade group			<0·001
GG1	NA	1·00	
GG2	Ref.		
GG3	1·14 (0·56-2·33)	0·72	
GG4	2·47 (0·71-8·59)	0·16	
GG5	4·52 (2·22-9·20)	<0·001	
Extraprostatic extension			0·058
No	Ref.		
Yes	1·73 (0·98-3·05)	0·058	
Seminal vesicle invasion			0·053
No	Ref.		
Yes	1·82 (0·99-3·36)	0·053	
Surgical margins			0·014
Free	Ref.		
Not free	1·96 (1·14-3·36)	0·014	
Lymph node metastasis			0·38
No	Ref.		
Yes	1·54 (0·59-4·04)	0·38	

***Supporting Table 19**: Multivariable analysis, prostate cancer, Oslo University Hospital 2*

Feature	HR (95% CI)	P-value feature level	P-value Anova
Mitotic figures per mm^2 (log_2-transformed)	1·20 (0·79-1·83)	0·39	0·39
Age	1·00 (0·96-1·05)	0·86	0·86
Preoperative PSA (log_2-transformed)	1·39 (1·03-1·86)	0·030	0·030
Gleason grade group			0·015
GG1	0·78 (0·27-2·23)	0·64	
GG2	Ref.		
GG3	2·27 (1·32-3·90)	0·003	
GG4	2·72 (1·24-5·99)	0·013	
GG5	3·14 (0·68-14·49)	0·14	
Extraprostatic extension			0·036
No	Ref.		
Yes	1·65 (1·03-2·64)	0·036	
Seminal vesicle invasion			0·092
No	Ref.		
Yes	1·64 (0·92-2·93)	0·092	
Surgical margins			0·048
Free	Ref.		
Not free	1·65 (1·01-2·69)	0·048	
Lymph node metastasis			0·093
No	Ref.		
Yes	2·16 (0·88-5·29)	0·093	

***Supporting Table 20**: Multivariable analysis, prostate cancer, Vestfold Hospital Trust*

Feature	HR (95% CI)	P-value feature level	P-value Anova
Mitotic figures per mm^2 (log_2-transformed)	1·03 (0·78-1·36)	0·85	0·85
Age	1·00 (0·97-1·04)	0·81	0·81
Preoperative PSA (log_2-transformed)	1·32 (1·04-1·67)	0·020	0·020
Gleason grade group			<0·001
GG1	0·44 (0·22-0·89)	0·021	
GG2	Ref.		
GG3	1·91 (1·15-3·18)	0·013	
GG4	1·94 (0·97-3·87)	0·062	
GG5	3·52 (2·01-6·16)	<0·001	
Pathological T-stage			0·14
pT2	Ref.		
pT3	1·37 (0·90-2·06)	0·14	
Surgical margins			0·066
Free	Ref.		
Not free	1·44 (0·98-2·14)	0·066	
Lymph node metastasis			0·003
No	Ref.		
Yes	9·14 (2·09-40·01)	0·003	

***Supporting Table 21**: Multivariable analysis, prostate cancer, university hospitals of Northern Norway*

Feature	HR (95% CI)	P-value feature level	P-value Anova
Mitotic figures per mm^2 (log_2-transformed)	0·91 (0·55-1·53)	0·73	0·73
Age	1·05 (1·00-1·09)	0·038	0·038
Sex			0·31
Female	Ref.		
Male	1·88 (0·55-6·45)	0·31	
WHO grade (2004)			0·017
Low grade	Ref.		
High grade	4·47 (1·31-15·29)	0·017	
Multifocal			0·064
No	Ref.		
Yes	2·36 (0·95-5·88)	0·064	
Urothelial carcinoma in situ			0·12
No	Ref.		
Yes	2·19 (0·81-5·93)	0·12	

Supporting Table 22*: Multivariable analysis, bladder cancer, Stavanger University Hospital*

Feature	HR (95% CI)	P-value feature level	P-value Anova
Mitotic figures per mm^2 (log_2-transformed)	1·28 (0·89-1·86)	0·18	0·18
Age	0·97 (0·94-1·00)	0·054	0·054
Oestrogen receptor status			0·64
Positive	Ref.		
Borderline	0·54 (0·12-2·38)	0·41	
Negative	0·73 (0·27-1·95)	0·53	
Progesterone receptor status			0·083
Positive	Ref.		
Borderline	1·74 (0·76-3·95)	0·19	
Negative	0·53 (0·19-1·48)	0·23	
HER2 status			0·39
Positive	Ref.		
Borderline	1·62 (0·48-5·42)	0·43	
Negative	1·65 (0·74-3·70)	0·22	
Nottingham grade			0·25
3-5	Ref.		
6-7	2·29 (0·85-6·20)	0·10	
8-9	2·05 (0·56-7·46)	0·28	
Nuclear atypia			0·96
Mild variation	NA	1·00	
Moderate variation	Ref.		
Marked variation	0·90 (0·44-1·84)	0·78	
Tumour diameter			0·11
≤ 2 cm	Ref.		
> 2 cm	1·74 (0·88-3·44)	0·11	

***Supporting Table 23**: Multivariable analysis, breast cancer 1990-1998, Stavanger University Hospital*

Feature	HR (95% CI)	P-value feature level	P-value Anova
Mitotic figures per mm^2 (log_2-transformed)	1·08 (0·64-1·84)	0·76	0·76
Age	1·03 (0·99-1·07)	0·098	0·098
Oestrogen receptor status			0·48
Positive	Ref.		
Negative	0·53 (0·09-3·00)	0·48	
Progesterone receptor status			0·75
Positive	Ref.		
Borderline	NA	NA	
Negative	1·28 (0·29-5·70)	0·75	
Nottingham grade			0·17
3-5	Ref.		
6-7	0·33 (0·07-1·60)	0·17	
8-9	0·84 (0·16-4·45)	0·84	
Nuclear atypia			1·00
Mild variation	NA	1·00	
Moderate variation	Ref.		
Marked variation	1·05 (0·36-3·07)	0·93	
Tumour diameter			0·47
≤ e2 cm	Ref.		
> 2 cm	1·37 (0·58-3·27)	0·47	
Lymph node metastasis			0·003
Negative	Ref.		
Positive	5·09 (1·73-14·97)	0·003	

Supporting Table 24*: Multivariable analysis, breast cancer 2000-2004, Stavanger University Hospital*

Feature	HR (95% CI)	P-value feature level	P-value Anova
Mitotic figures per mm^2 (log$_2$-transformed)	1·00 (0·84-1·20)	0·96	0·96
Age			<0·001
<60	Ref.		
≥60	2·52 (1·66-3·83)	<0·001	
FIGO stage			<0·001
IA	Ref.		
IB	1·36 (0·95-1·94)	0·093	
II	3·86 (2·48-6·00)	<0·001	
IIIA	3·50 (1·87-6·53)	<0·001	
IIIB	8·90 (3·45-22·94)	<0·001	
III	3·51 (2·31-5·35)	<0·001	
Histological type and differentiation			<0·001
Endometrioid and grade 1	Ref.		
Endometrioid and grade 2	1·57 (1·06-2·31)	0·023	
Endometrioid and grade 3	1·68 (1·03-2·72)	0·037	
Mixed without c/s and grade 1	0·79 (0·29-2·21)	0·66	
Mixed without c/s and grade 2	2·35 (0·85-6·54)	0·10	
Mixed without c/s and grade 3	4·72 (1·45-15·43)	0·010	
Mucinous carcinoma	1·07 (0·15-7·80)	0·94	
Clear cell adenocarcinoma	1·45 (0·60-3·48)	0·41	
Serous carcinoma	2·57 (1·59-4·14)	<0·001	
Mixed with c/s	1·65 (0·91-3·01)	0·100	
Undifferentiated carcinoma	2·00 (0·47-8·48)	0·35	
Unclassifiable carcinoma	2·11 (0·63-7·06)	0·22	
Neuroendocrine tumour	1·75 (0·52-5·85)	0·36	
Carsinosarcoma	4·34 (2·49-7·58)	<0·001	
Lymphovascular space invasion			0·001
No	Ref.		
Yes	1·62 (1·21-2·17)	0·001	
Adjuvant treatment			<0·001
No	Ref.		
Yes	0·54 (0·39-0·75)	<0·001	

Supporting Table 25*: Multivariable analysis, endometrial cancer, Oslo University Hospital*

Abbreviations: c/s: clear cell carcinoma or serous carcinoma

		HR (95% CI)	p
Mitotic count (average)	log_2(1+mitosis/mm^2) increment	1·26 (1·03-1·54)	0·027
Age	10-year increment	1·17 (0·99-1·38)	0·061
Sex	Male vs· Female	1·09 (0·83-1·42)	0·53
Smoking status	Former or current vs· Never	0·99 (0·60-1·64)	0·96
Pathological stage			<0·0001
	I	Ref.	
	II	1·72 (1·27-2·34)	
	III	2·69 (1·90-3·80)	
	IV	5·34 (2·26-12·60)	
ECOG performance status	≥2 vs· <2	1·80 (1·01-3·21)	0·044
Histological type			0·0022
	Adenocarcinoma	Ref.	
	Squamous cell carcinoma	0·68 (0·49-0·96)	
	Large cell and undifferentiated carcinoma	1·52 (0·83-2·80)	
	Carcinoid tumour	0·13 (0·02-0·96)	
	Other	1·78 (0·90-3·52)	

Supporting Table 26*: Multivariable analysis, lung cancer, Oslo University Hospital*

		HR (95% CI)	p
Mitotic count	$\log_2$(1+mitosis/mm^2) increment	1·31 (1·09-1·58)	0·0045
Age	10-year increment	1·05 (0·90-1·23)	0·50
Sex	Male vs· Female	1·47 (1·07-2·01)	0·017
Smoking status	Former or current vs· Never	0·42 (0·22-0·80)	0·0084
Pathological stage			<0·0001
	I	Ref.	
	II	1·74 (1·22-2·47)	
	III	4·42 (3·14-6·20)	
ECOG performance status	≥2 vs· <2	1·52 (0·82-2·81)	0·18
Histological type			0·012
	Adenocarcinoma	Ref.	
	Squamous cell carcinoma	0·63 (0·46-0·85)	
	Large cell and undifferentiated carcinoma	0·00 (0·00-∞)	
	Other	2·30 (0·56-9·45)	

Supporting Table 27*: Multivariable analysis, lung cancer, University hospitals of Northern Norway*

Feature	HR (95% CI)	P-value feature level	P-value Anova
Mitotic figures per mm^2 (log_2-transformed)	1·11 (0·93-1·31)	0·26	0·26
Age	1·01 (0·99-1·02)	0·28	0·28
Pathological T stage			<0·001
pT1	Ref.		
pT2	NA	0·99	
pT3	1·45 (0·20-10·58)	0·71	
pT4	4·16 (0·57-30·24)	0·16	
Pathological N stage			<0·001
pN0	Ref.		
pN1	1·75 (1·22-2·50)	0·002	
pN2	4·35 (3·09-6·12)	<0·001	
Tumour location			0·67
Proximal	Ref.		
Distal	1·15 (0·85-1·54)	0·37	
Rectum	1·07 (0·78-1·46)	0·70	

***Supporting Table 28**:Multivariable analysis, colorectal cancer, Cheltenham General Hospital*

Feature	HR (95% CI)	P-value feature level	P-value Anova
Mitotic figures per mm^2 (log_2-transformed)	0·83 (0·66-1·03)	0·094	0·094
Age	1·00 (0·99-1·02)	0·82	0·82
Pathological T stage			<0·001
pT1	Ref.		
pT2	2·80 (0·35-22·37)	0·33	
pT3	2·06 (0·28-14·91)	0·48	
pT4	4·35 (0·60-31·49)	0·15	
Pathological N stage			<0·001
pN0	Ref.		
pN1	2·09 (1·29-3·38)	0·003	
pN2	7·66 (4·82-12·15)	<0·001	
Tumour location			0·36
Proximal	Ref.		
Distal	0·78 (0·54-1·11)	0·16	
Rectum	0·82 (0·48-1·38)	0·45	

Supporting Table 29*: Multivariable analysis, colorectal cancer, QUASAR 2*

Feature	HR (95% CI)	P-value feature level	P-value Anova
Mitotic figures per mm^2 (log_2-transformed)	1·41 (1·00-1·99)	0·049	0·049
Age	1·02 (1·00-1·04)	0·045	0·045
Gender			0·059
Female	Ref.		
Male	1·53 (0·98-2·39)	0·059	
Synchronous/metachronous			0·91
Synchronous	Ref.		
Metachronous	1·03 (0·61-1·73)	0·91	
Size of largest lesion			0·13
≤50mm	Ref.		
>50mm	1·44 (0·90-2·32)	0·13	
Number of metastases			0·052
1	Ref.		
>1	1·57 (1·00-2·47)	0·052	
Pathological T stage of primary tumour			0·82
pT1	Ref.		
pT2	0·73 (0·23-2·25)	0·58	
pT3	0·64 (0·22-1·85)	0·41	
pT4	0·60 (0·19-1·88)	0·38	
Pathological N stage of primary tumour			0·16
pN0	Ref.		
pN1	1·07 (0·64-1·76)	0·81	
pN2	1·56 (0·95-2·57)	0·081	
Location of primary tumour			0·028
Right	Ref.		
Left	0·56 (0·33-0·94)	0·028	
Extra hepatic disease			0·12
No	Ref.		
Yes	1·67 (0·87-3·22)	0·12	
Neoadjuvant therapy			0·74
No	Ref.		
Yes	1·08 (0·70-1·65)	0·74	
Adjuvant therapy			0·29
No	Ref.		
Yes	1·27 (0·82-1·95)	0·29	

Supporting Table 30*: Multivariable analysis, colorectal cancer liver metastases, Liverpool*

	Representation	HR (95% CI)	P-value	Concordance index
Original representation uterine sarcomas	Mitoses per mm^2 (log2-transformed)	1·67 (1·49-1·87)	<0·001	0·67
Max density $2mm^2$ uterine sarcomas	Mitoses per mm^2 (log2-transformed)	1·60 (1·42-1·80)	<0·001	0·66
Manual counting, uterine sarcomas	Mitotic figures per 10HPF (log2-transformed)	1·43 (1·32-1·56)	<0·001	0·66
Original representation breast cancer 1990-1998	Mitoses per mm^2 (log2-transformed)	1·70 (1·31-2·21)	<0·001	0·67
Max density $2mm^2$ breast cancer 1990-1998	Mitoses per mm^2 (log2-transformed)	1·59 (1·24-2·03)	<0·001	0·67
Manual counting, breast cancer 1990-1998	Mitotic figures per 10HPF (log2-transformed)	1·42 (1·21-1·67)	<0·001	0·70
Original representation breast cancer 2000-2004	Mitoses per mm^2 (log2-transformed)	1·34 (1·05-1·70)	0·018	0·64
Max density $2mm^2$ breast cancer 2000-2004	Mitoses per mm^2 (log2-transformed)	1·30 (1·04-1·61)	0·021	0·63
Manual counting, breast cancer 2000-2004	Mitotic figures per 10HPF (log2-transformed)	1·20 (1·03-1·40)	0·019	0·64

Supporting Table 31*: Prognostic impact of automatic mitotic figure counting in the entire tumour region, in the most mitotic figure dense 2 mm^2 hotspot, and manually counted mitotic figures in the uterine sarcomas testset and the two breast cancer datasets from Stavanger University Hospital.*

Validation dataset	Endpoint	HR (95% CI)	P-value
Colorectal cancer Cheltenham	Overall survival	0·94 (0·82 - 1·08)	0·367
Colorectal cancer QUASAR2	Overall survival	0·82 (0·68 - 0·99)	0·040
Liver metastases Liverpool	Recurrence	1·08 (0·85 - 1·37)	0·516

Supporting Table 32*: Mitotic figure counts analysed with other endpoints*

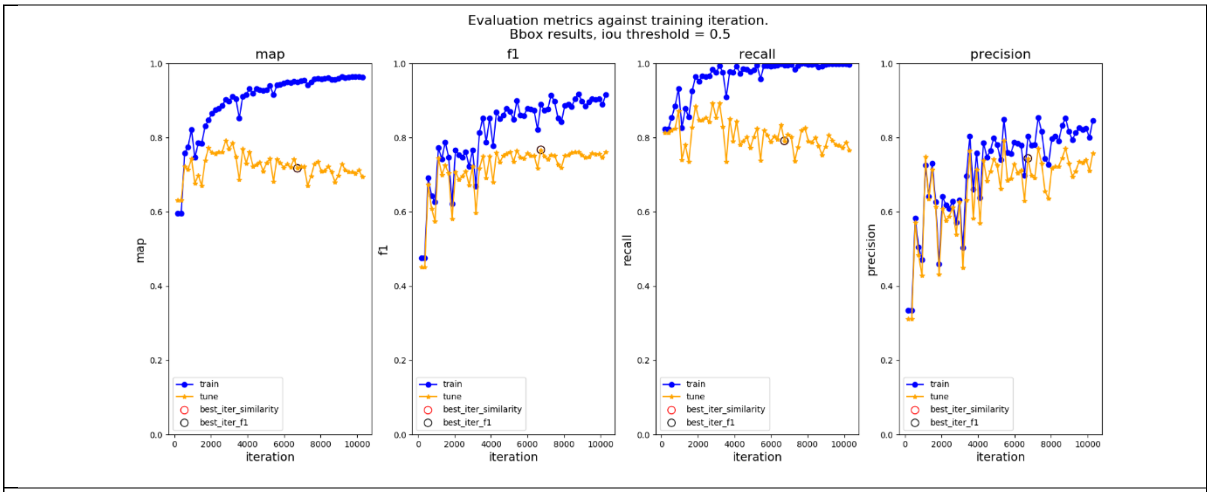
Evaluation metrics against training iteration.
Bbox results, iou threshold = 0.5
map
f1
recall
precision
iteration
train
tune
best_iter_similarity
best_iter_f1

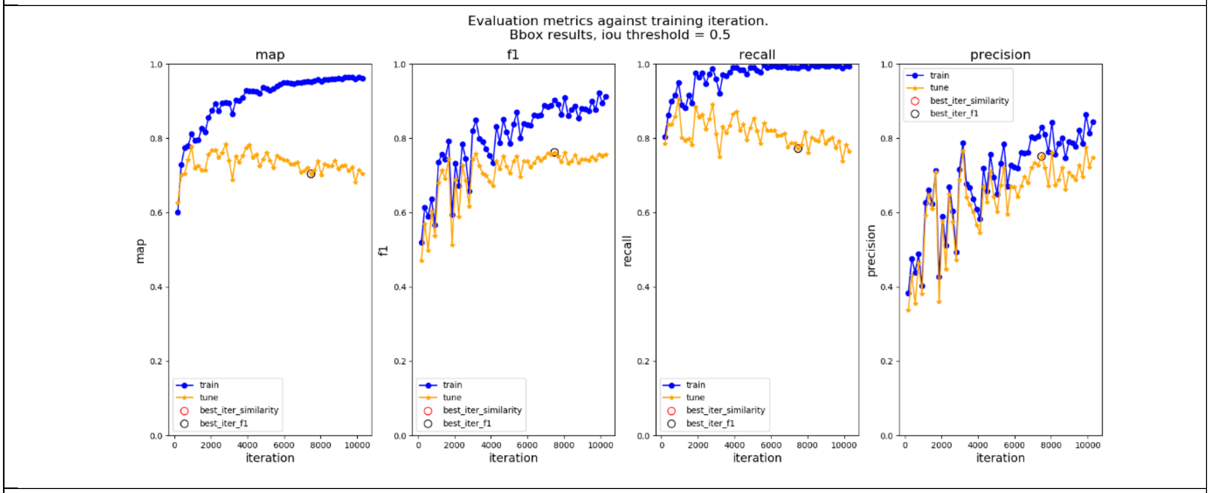
Evaluation metrics against training iteration.
Bbox results, iou threshold = 0.5
map
f1
recall
precision
iteration
train
tune
best_iter_similarity
best_iter_f1

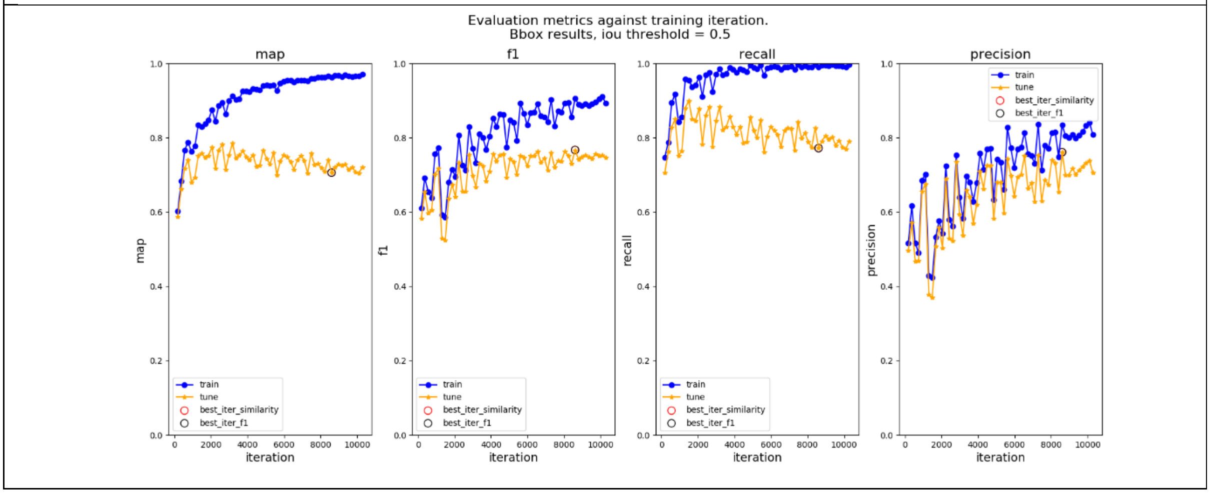
Evaluation metrics against training iteration.
Bbox results, iou threshold = 0.5
map
f1
recall
precision
iteration
train
tune
best_iter_similarity
best_iter_f1

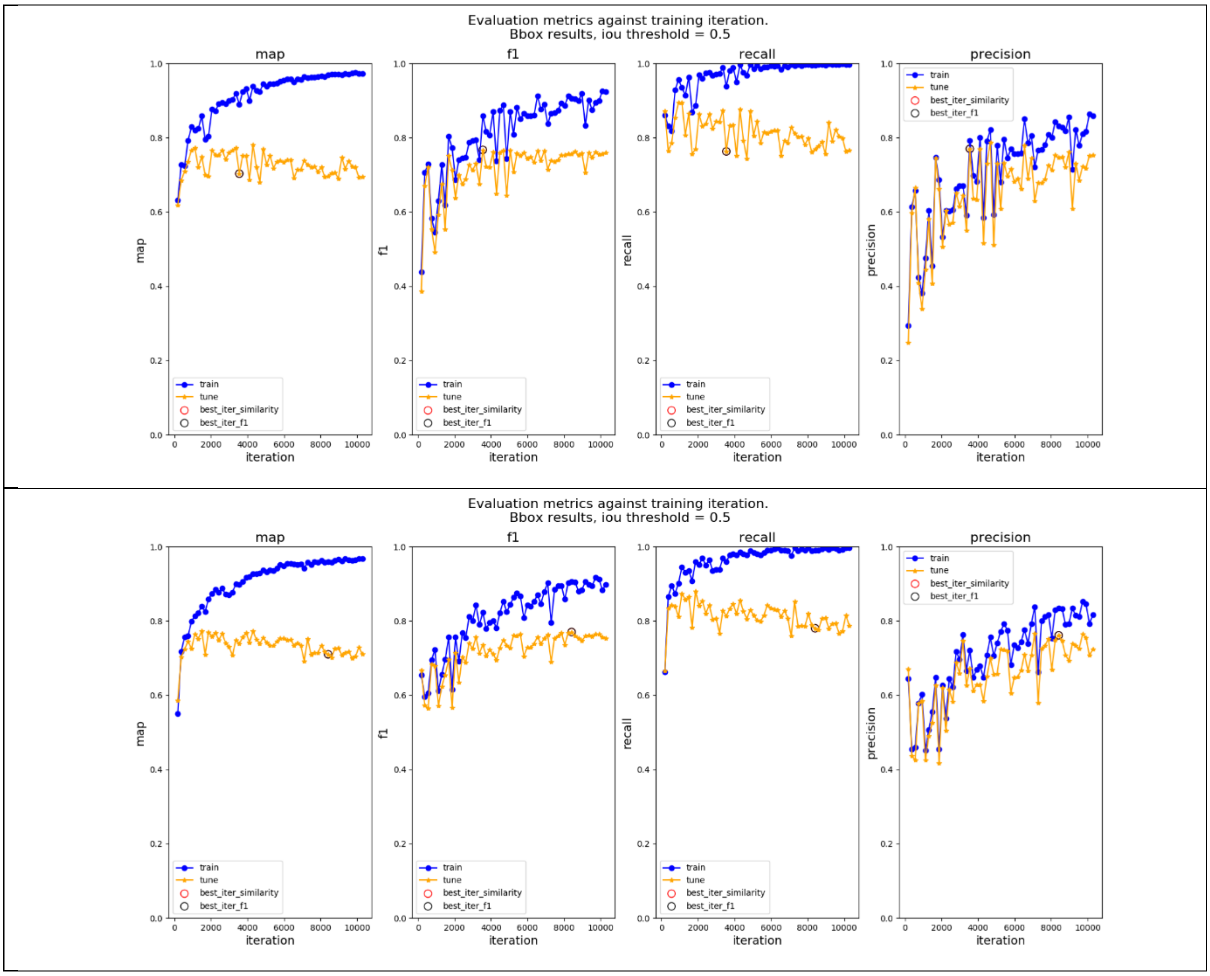

Supporting Figure 1: *Performance metrics vs training iteration for the 5 independently trained Mask RCNN models. The iteration with the highest f1-score was selected and is indicated with a circle in the plots.*

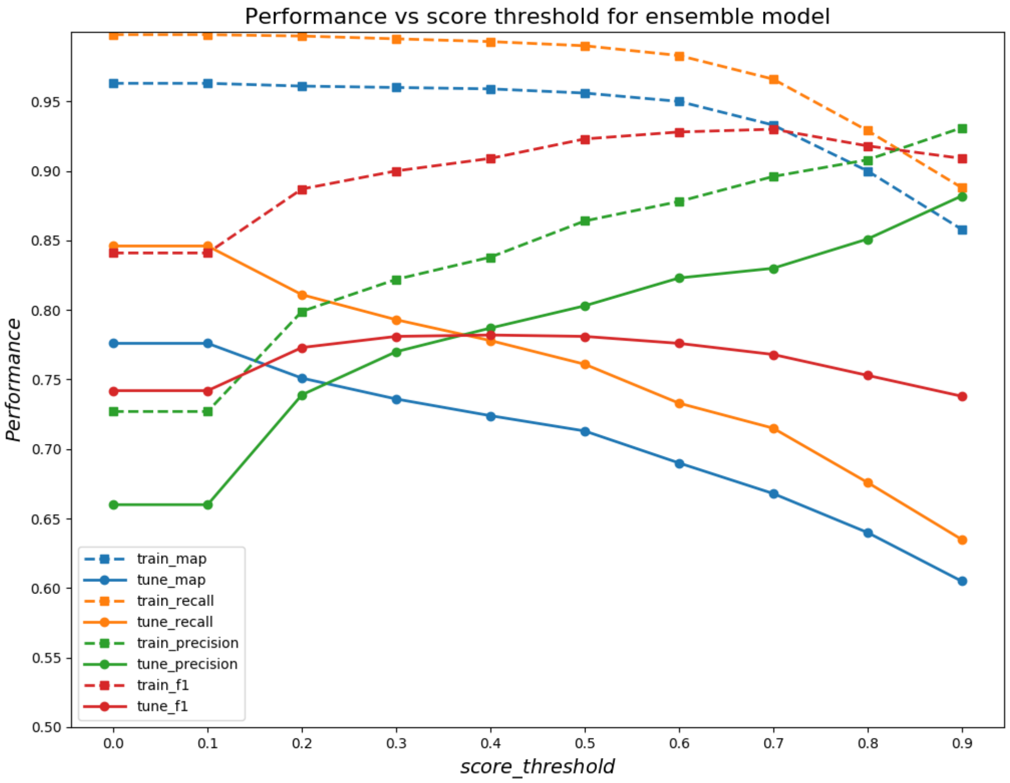

Supporting Figure 2: *Performance metrics in the train and tune datasets vs score threshold for the ensemble model. A score threshold of 0.4 was selected. Detections with ensemble score below 0.4 were excluded.*

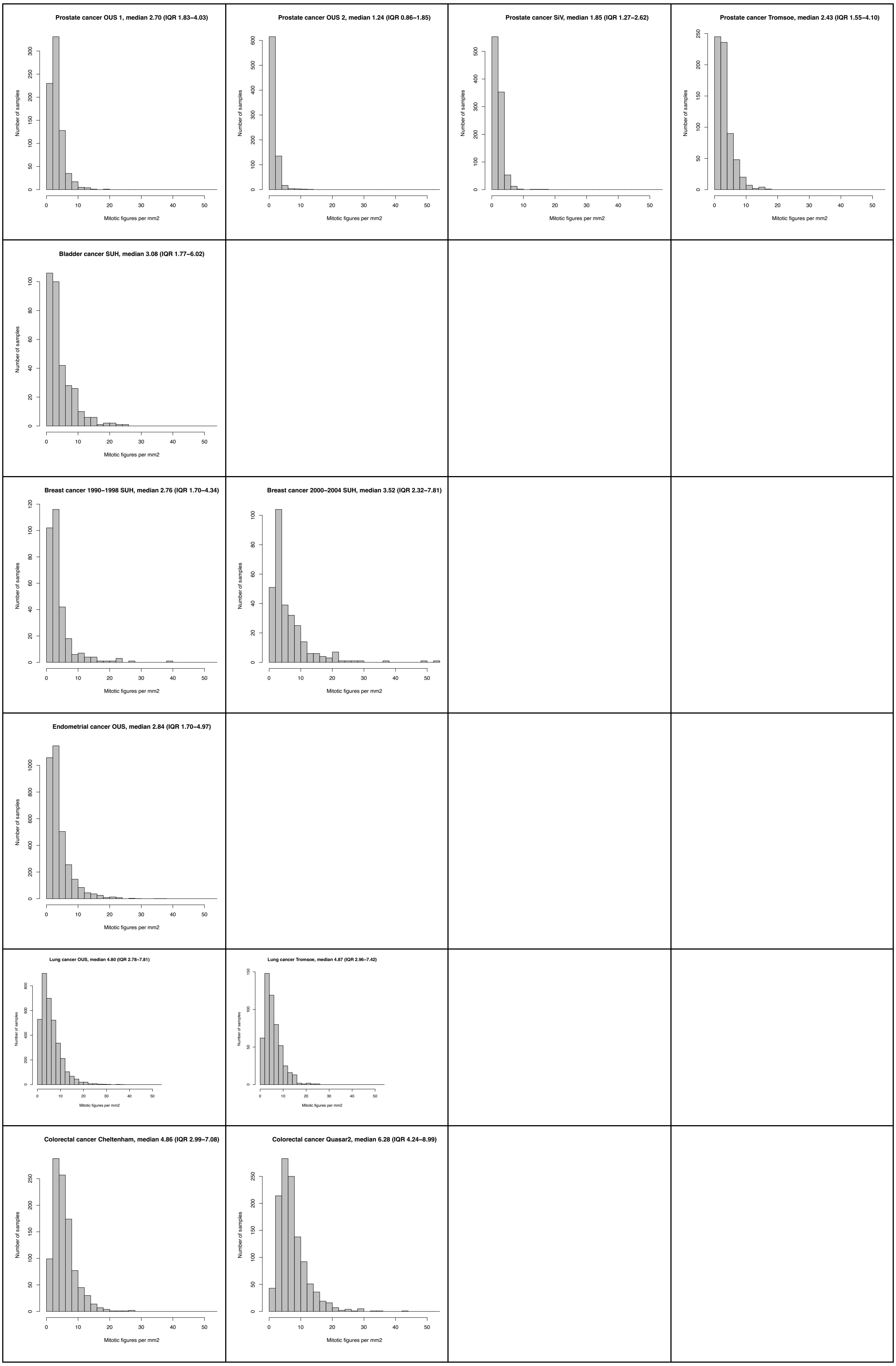
Prostate cancer OUS 1, median 2.70 (IQR 1.83–4.03)
Prostate cancer OUS 2, median 1.24 (IQR 0.86–1.85)
Prostate cancer SiV, median 1.85 (IQR 1.27–2.62)
Prostate cancer Tromsoe, median 2.43 (IQR 1.55–4.10)
Bladder cancer SUH, median 3.08 (IQR 1.77–6.02)
Breast cancer 1990–1998 SUH, median 2.76 (IQR 1.70–4.34)
Breast cancer 2000–2004 SUH, median 3.52 (IQR 2.32–7.81)
Endometrial cancer OUS, median 2.84 (IQR 1.70–4.97)
Lung cancer OUS, median 4.80 (IQR 2.78–7.81)
Lung cancer Tromsoe, median 4.87 (IQR 2.96–7.42)
Colorectal cancer Cheltenham, median 4.86 (IQR 2.99–7.08)
Colorectal cancer Quasar2, median 6.28 (IQR 4.24–8.99)
Number of samples
Mitotic figures per mm2

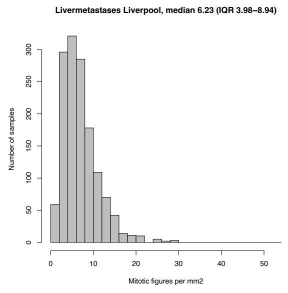

***Supporting Figure 3**: Histograms of detected mitotic figures per mm2 in the validation datasets*

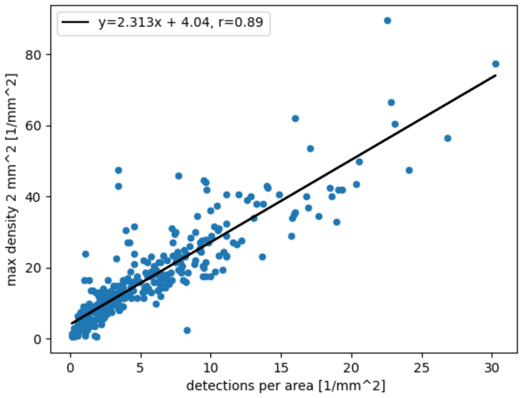

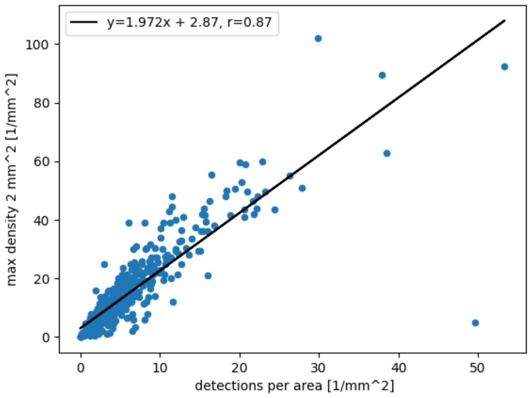

Supporting Figure 4: *Detected mitotic figures per mm^2 in the most mitotic figure dense 2 mm^2 circle shaped hotspots vs in the entire tumour region in uterine sarcomas test dataset (left) and the two breast cancer datasets (right).*

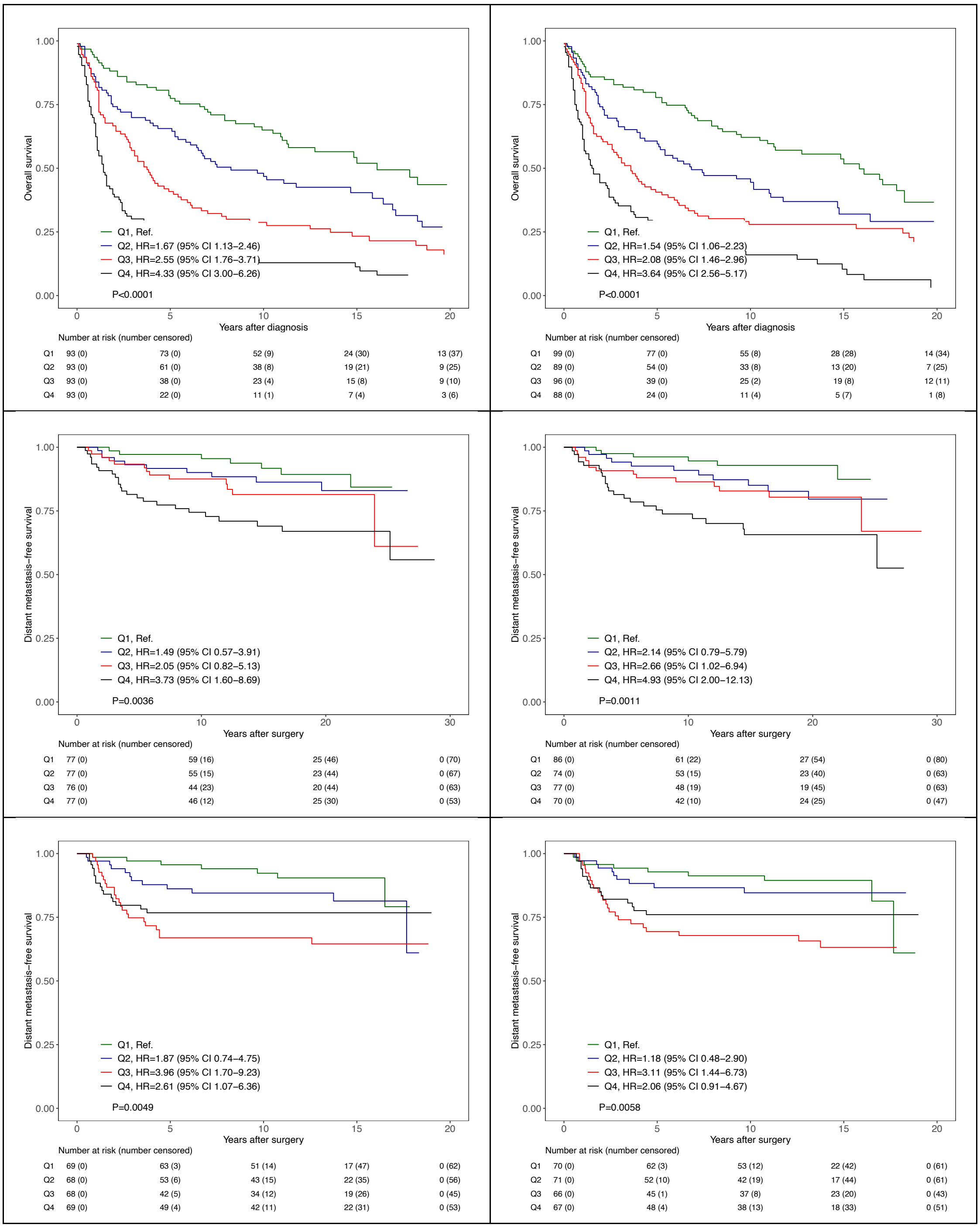

Supporting Figure 5: *Kaplan-Meier survival curves for original and hotspot representation for mitotic figures in the uterine sarcomas testset and the two breast cancer datasets from Stavanger University Hospital.*

Protocol for the validation of a deep learning system for automatic mitotic figure counting

Status at last amended

This protocol was last modified on 23th of November 2021, prior to all investigations that could reveal associations between automatic mitotic figure counts and clinical outcome in the validation cohorts. At that time the haematoxylin and eosin-stained tissue slides from the validation cohorts had been scanned and analysed blinded to the clinical outcome.

System

The proposed deep learning system for detection of mitotic figures was based on the Mask R-CNN implementation in the Detectron2 framework (https://github.com/facebookresearch/detectron2). Mask R-CNN [1] is an instance segmentation network that takes an image as input and outputs object detections, including segmentations, bounding boxes around the detected objects and corresponding class labels. In the task of detecting mitotic figures in images, we have only one class label, i.e. the mitotic figure class. The system segments the detected objects and assigns a score to each object representing the probability of belonging to the mitotic figure class.

The system has two stages where the first stage generates object proposals in the shape of bounding boxes, i.e. rectangular candidate regions potentially containing mitotic figures. The second stage predicts the class label of the proposed object, refines the bounding box and generates a segmentation mask on pixel level for the object. Both of these stages are connected to a backbone convolutional neural network; we used ResNet-50-FPN (FPN=Feature Pyramid Network) from the above referred github repository, pretrained on the ImageNet dataset [2]. Default hyperparameters were used unless otherwise specified below. Training was performed on a standard computer with Linux operating system and 2 Nvidia Titan RTX GPU cards with 24Gb memory each. A batch size of 4 images was used.

Images of size 1024x1024 pixels with associated mitotic figure ground truth information were used for training. Normalisation of each tile image was performed with the HE-normalisation method by Macenko et al. [3] and was applied on a tile level. HE-normalisation was performed after the distortions described in the following section.

Distortion was applied to each tile used in the network during training. All distortions were done using *torchvision transforms* (https://pytorch.org/docs/stable/torchvision/transforms.html). The following distortions were performed in the listed order:

1. The image was flipped horizontally with a probability of 0.5, or remained unflipped (also with a probability of 0.5).
2. The image was flipped vertically with a probability of 0.5, or remained unflipped (also with a probability of 0.5).
3. The image was converted from red, green and blue (RGB) colour space to hue, saturation and value (HSV) colour space, the hue channel was cyclically shifted with a random value drawn from the uniform distribution on [-0.2, 0.2], and the image was converted back to RGB colour space, all using the function

torchvision.transforms.functional.adjust_hue. For reference, note that a cyclical shift of -0.5 or 0.5 gives an image with complementary colours.

4. Saturation was adjusted with a saturation factor randomly drawn from the uniform distribution on [0.6, 1.4] using the function *torchvision.transforms.functional.adjust_saturation*.
5. Brightness was adjusted with a brightness factor randomly drawn from the uniform distribution on [0.8, 1.2] using the function *torchvision.transforms.functional.adjust_brightness*.
6. Contrast was adjusted with a contrast factor randomly drawn from the uniform distribution [0.6, 1.4] using the function *torchvision.transforms.functional.adjust_contrast*.

For the anchor generator, i.e. object proposal generator, we used angles of [-90, 0, 90], aspect ratios of [0.5, 1, 2] and sizes [64].

For ROI_HEADS, we set the following parameters:

- SCORE_THRESH_TEST: 0.7
- BATCH_SIZE_PER_IMAGE: 256
- POSITIVE_FRACTION: 0.50
- NUM_CLASSES: 1

For ROI_BOX_HEAD, we set the following parameters:

- POOLER_RESOLUTION: 7
- POOLER_SAMPLING_RATIO: 2

For ROI_MASK_HEAD, we set the following parameters:

- POOLER_RESOLUTION: 14
- POOLER_SAMPLING_RATIO: 2

For PIXEL_MEAN, we used the mean values of B, G and R channels of the HE-normalised images included in the training set.

PIXEL_MEAN = [169.17, 116.28, 184.31].

We trained the network for 55 epochs (10285 iterations). A base learning rate of 0.002 was used. We used a linear warmup with 200 iterations and a warmup factor of 0.001. The learning rate was halved every 10th epoch.

The maximum number of detections to return per image during inference was set to 500 (default is 100 based on the limit established for the COCO datasets).

During training, an object proposal was considered, by the region proposal network, a true object if the Intersection over Union (IoU) with any ground truth object was at least 0.7, and a background object if the IoU was maximum 0.3. The final automatic detection was considered correct if and only if the IoU with a ground truth object was more than 0.5 and the predicted class was the same as the ground truth class. Recall (the proportion of the ground truth mitotic figures detected by the system) and precision (the proportion of the automatic mitotic figure detections that were correct) were calculated for every epoch/187th model iteration in the tuning set (described below) and used in the evaluation of model iterations. To identify candidate models and select the best, we required models to have precision ∈ (recall / (1 + t), recall / (1 - t)), t: 0.1. We then used

the highest F1-score (2*precision*recall/(precision+recall)) to select the best model among the acceptable solutions identified with the precision interval.

Five independent instances of the deep learning network were trained for 10285 iterations, corresponding to presenting all images to the network 55 times. A checkpoint model was saved after each epoch. The best training iteration for each model was identified from these 55 saved checkpoints as described in the previous section. The five selected individual models were combined in an ensemble model. Predictions from two models were considered overlapping when the intersection over union (IoU) of the bounding boxes was higher than 0.5. The bounding box and segmentation of the highest scoring individual model, and the mean prediction score from the overlapping predictions were used to represent the result. Models without predictions were represented with a score of 0 in the calculation of mean score. The optimal threshold for mean prediction score from the ensemble model was identified with the same optimality criterion as used for the single models, i.e. select the model with the highest F1-score from models within the acceptable precision interval.

Edge detections

Mitotic figures that span more than one image tile are rare, but may be counted more than once as the deep learning system analyses each image independently. To compensate for this, we identified detections on the edges of neighbouring tiles and adjusted the mitotic figure counts correspondingly, described in the following. The test set (described below) was used to develop and adapt the method.

Two different thresholds were used to identify detections located between two tiles. One was used to find all tiles with detections close to the edges, and one to decide if two detections were likely discovering the same mitosis. These are demonstrated in Protocol Figure 1.

Mitosis detection was applied to merged tiles to obtain a ground truth that was used to evaluate whether the proposed double-counted edge detections were actually the same mitotic figures (see example in Protocol Figure 2).

All neighbouring tiles with detections close to their common edge were first identified. Then either the detections' y or x coordinates of both the upper left and lower right corners were compared, depending on whether the neighbour tiles were lying side by side or above each other, respectively. If any of the detections' corners were closer to each other than the threshold, these were classified as duplicates, and one of them was removed from the total count.

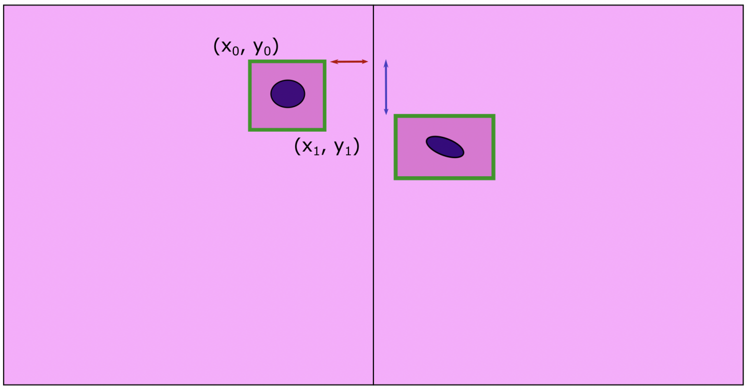

Protocol Figure 1: The red arrow represents the distance to edge threshold. The blue arrow represents the threshold used to consider if two detections along a common tile edge is the same mitosis.

To find the optimal threshold values, threshold combinations were applied in the uterine sarcomas test set and the updated mitosis counts were compared to the ground truth established with merged tiles. See examples of candidate double detections in Protocol Figure 3. The lowest sum of false negatives (i.e. missed true double detections) and false positives (i.e. wrong double detection predictions) was used to find an optimal solution. An optimum was found for the threshold combination [5, 32], i.e. consider neighbouring detections up to 5 pixels from the edge with up to 32 pixels displacement along the edge as the same mitotic figure. With this threshold combination, 342 edge detections out of 368 in the total test set were detected. This constitutes 0.14% of all mitotic figure detections in the test set.

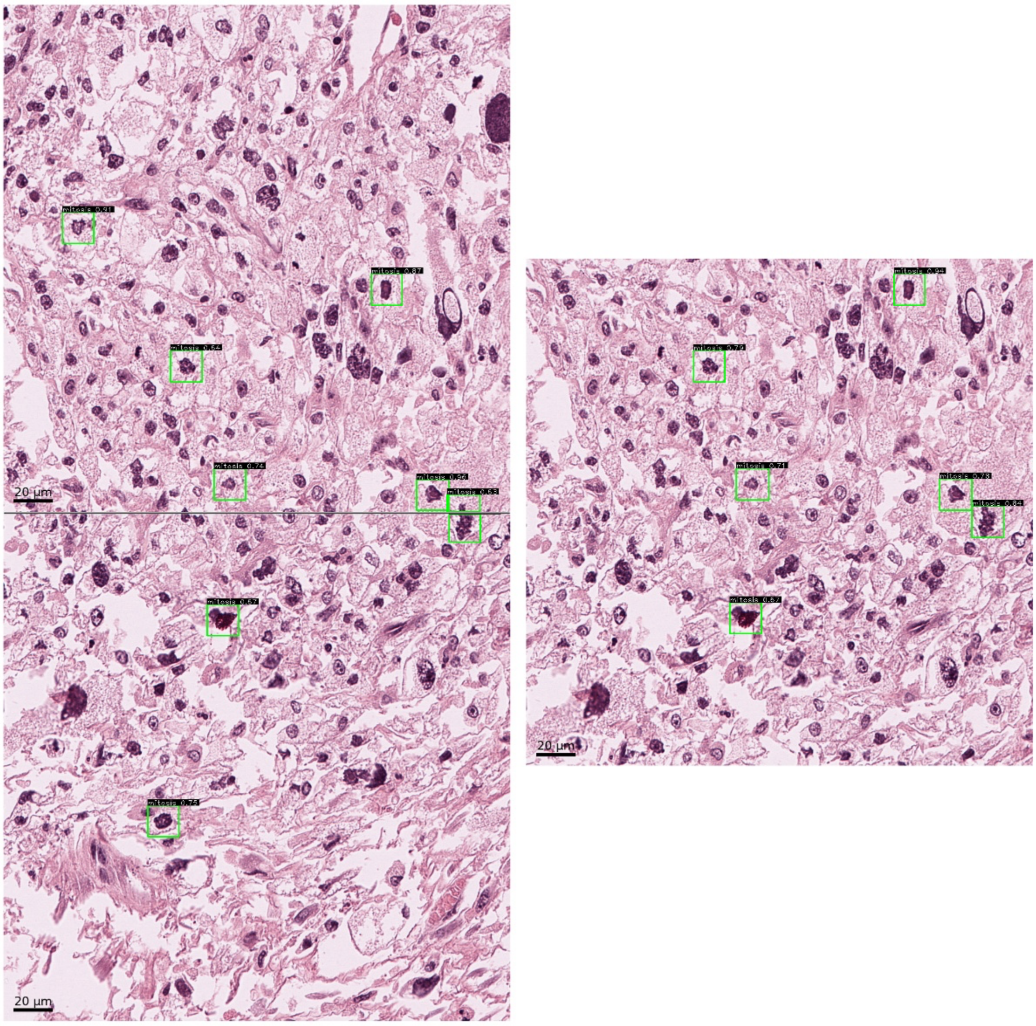

Protocol Figure 2: Left: Three detections are found along the common tile edge, where two of them are classified as duplicates. Right: The merged tile confirms that this classification is true as it only has two detections along the former edge.

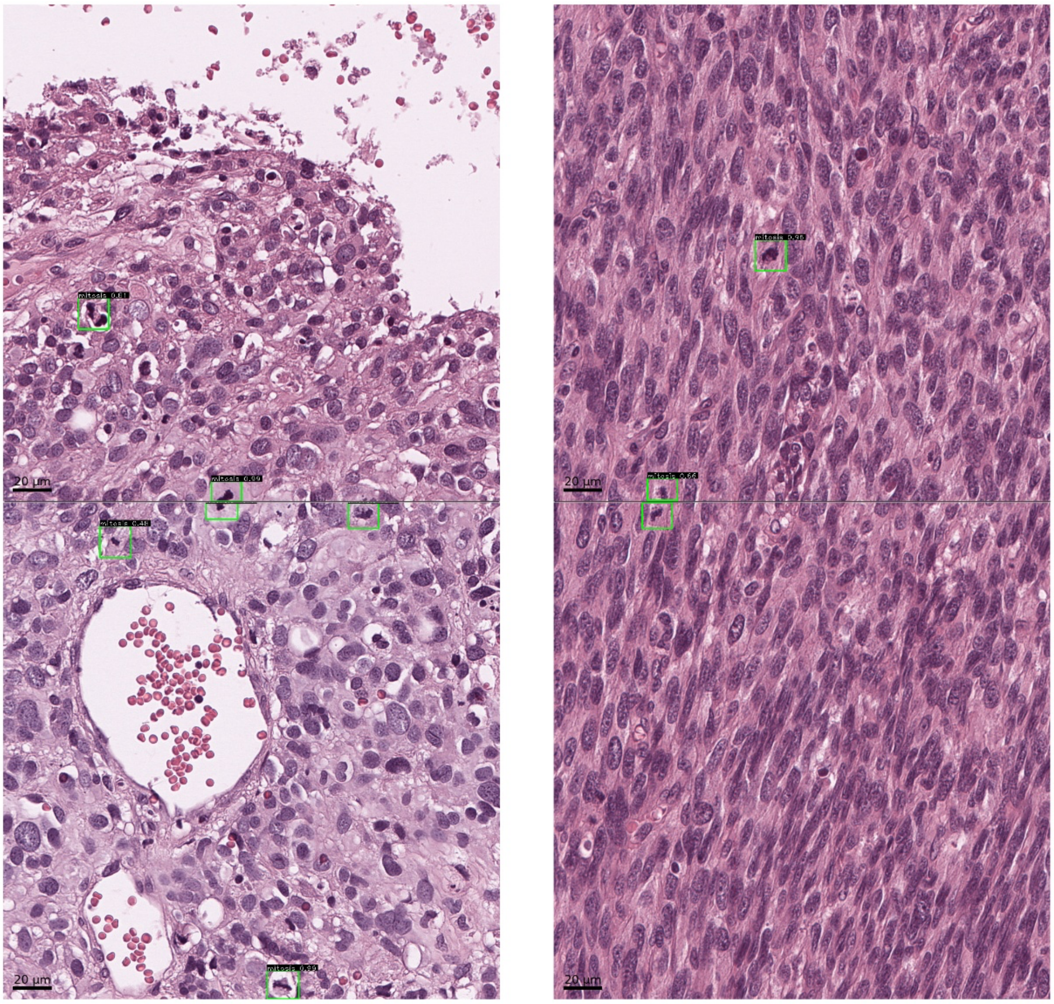

Protocol Figure 3: Examples of detections classified as duplicates. Left: true classification. Right: false classification.

Development dataset

Data from the "Tumor Proliferation Assessment Challenge 2016" and in particular the dataset for mitosis detection ("Auxiliary dataset: mitoses") was used to develop the deep learning system (https://tupac.tue-image.nl/node/3). This dataset consists of in total 73 scanned HE-stained breast cancer tissue sections with annotated mitotic figures that are the consensus of at least two pathologists. The first 23 tissue sections were previously included as part of the training dataset in the AMIDA13 challenge and were collected at the Department of Pathology at the University Medical Center in Utrecht, The Netherlands, and scanned with the Aperio ScanScope XT scanner. The remaining 50 tissue sections were from two different pathology centres in the Netherlands and each section was represented with one image region with area 2mm^2, scanned with the Leica SCN 400 scanner. Both scanners used the 40x objective lens setting. Mitotic figures were identified by a fixed-size box (64x64 pixels) with centre in the manually identified mitotic figure and the coordinates were provided in .csv-files. We split the development dataset in two partitions: a training partition intentionally containing 70% of the mitotic figures and a tuning partition with the remaining 30% mitotic figures, with the requirement that a scan cannot be included in both the training and tuning partitions. The partitioning resulted in a development dataset with 1147 mitotic figures (68%) from 38 scans in the training partition and 540 mitotic figures (32%) from 35 scans in the tuning partition.

Test dataset

A total population of uterine sarcomas in Norway diagnosed in the time period 1970-2000 was included in the study as a test cohort. The dataset has been described previously [4]. Uterine sarcomas are rare tumours with a generally poor outcome. Mitotic index is an established and strong prognostic marker in this tumour type, where the number of mitotic figures is manually counted in 10 high power fields (HPFs), corresponding to approximately 2mm^2. We used the uterine sarcomas dataset to evaluate the prognostic impact of candidate models. The primary analysis (described below), i.e. univariable Cox regression with the predicted number of mitotic figures per mm^2 (variable representation: log (variable + 1), log base 2) as covariate and overall survival as endpoint was calculated for all evaluated models. The concordance index [5] was used to evaluate accuracy and to rank the evaluated models, and the best performing model was selected for validation.

The patients' HE-slides were scanned on an Aperio AT2 scanner. In total 372 scans from 372 patients in the test cohort were available for analysis. Tumour area for analysis (annotations) were drawn manually by a pathologist. We evaluated models trained with normalised and not normalised training images and increasing levels of image augmentation in this dataset.

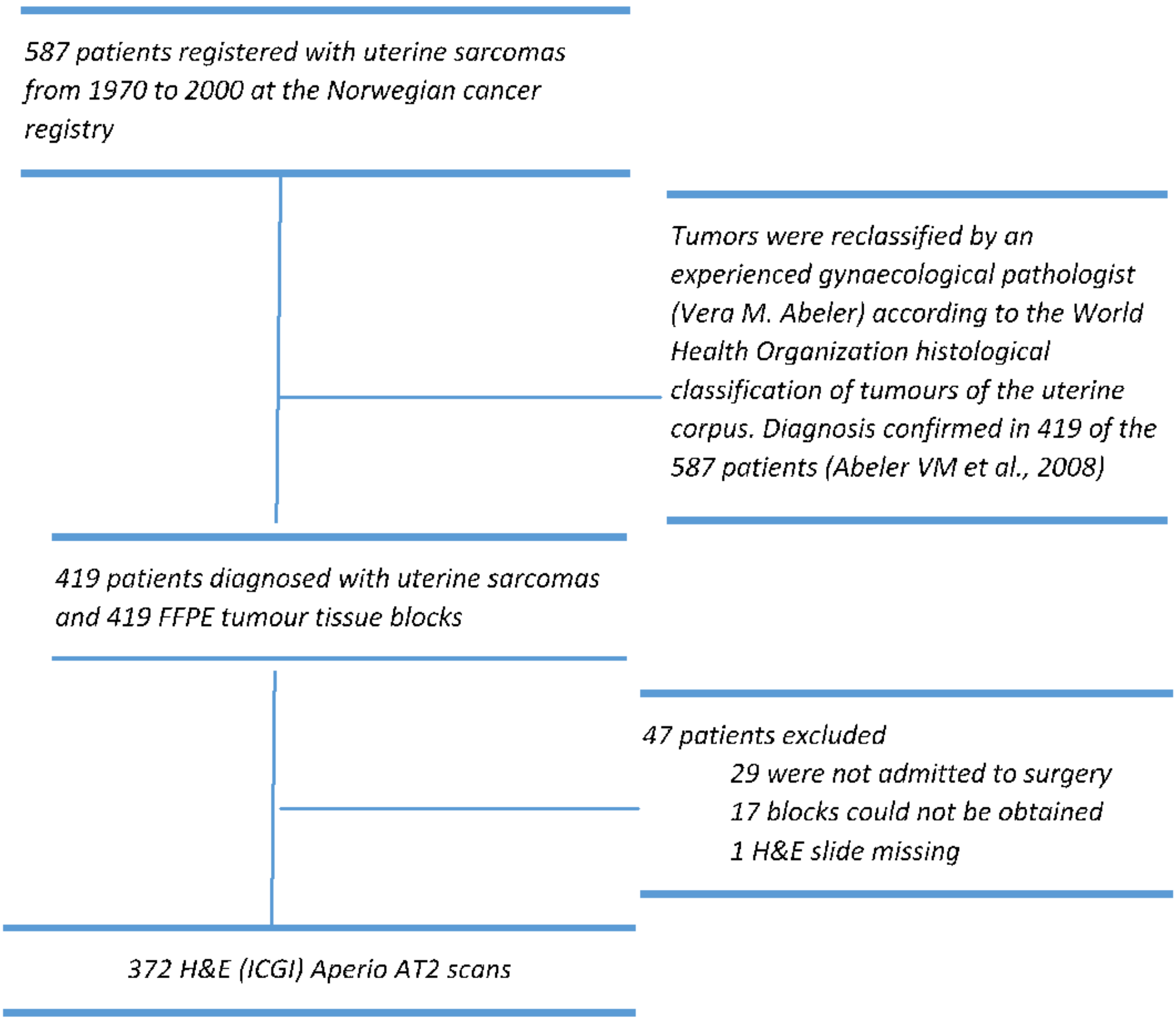

Protocol Figure 4: Inclusions and exclusions of patients and samples in the uterine sarcomas test set

Tumour annotation

The mitotic figure detection method requires samples to be annotated to identify the region for analysis. We have used manual tumour annotations drawn by a pathologist in this work. The annotations were drawn on scanned HE-sections using a software tool developed in-house. Areas containing malignant cells were annotated. In the case of breast carcinomas, large regions of intraductal carcinoma were not included in the annotation.

Background detection

Tiles (described below) not containing tissue (background tiles) were excluded from analysis. The background tiles were excluded using the following method:

1) Downsample whole slide image by a factor of 32.
2) Normalise using the method by Macenko et al. [3]
3) Convert from RGB to grayscale image.
4) Set a threshold:

   Threshold = $\bar{g} + 0.15p_{97}(0.5 - \frac{1}{1+ e^{-0.1(\bar{g}-0.95p_{97})}})$

   Where $\bar{g}$ is mean value in the grayscale image and p_{97} is the 97th percentile.
5) Mask = grayscale image < threshold. Mask is an image where foreground is True and background is False.

For each tile, find the corresponding area in the mask image. The tile is considered background if less than 25% of the pixels in the tile mask are foreground pixels.

Prediction on a new sample

Mitosis counting on a new sample requires the sample to be HE-stained and scanned with an Aperio AT2 scanner at full scanner resolution (40x objective lens). The tumour region of the scanned sample is annotated manually, split into non-overlapping image tiles of size 1024x1024 pixels, excluded for background tiles with method described above, normalised with the histology normalisation method by [3] with stain reference calculated on the individual tiles, and analysed by the trained deep learning model. The number of detected mitotic figures divided by total area (mm^2) is reported. If there are multiple tumour blocks for a patient, the total number of mitotic figures in all tumour blocks divided by the total area (mm^2) is reported.

External cohorts

The prognostic impact of the proposed method was evaluated in 13 validation cohorts that all were external to the development and test cohorts. The validation cohorts are described in the following section.

Breast cancer cohort 1990-1998 from Stavanger University Hospital

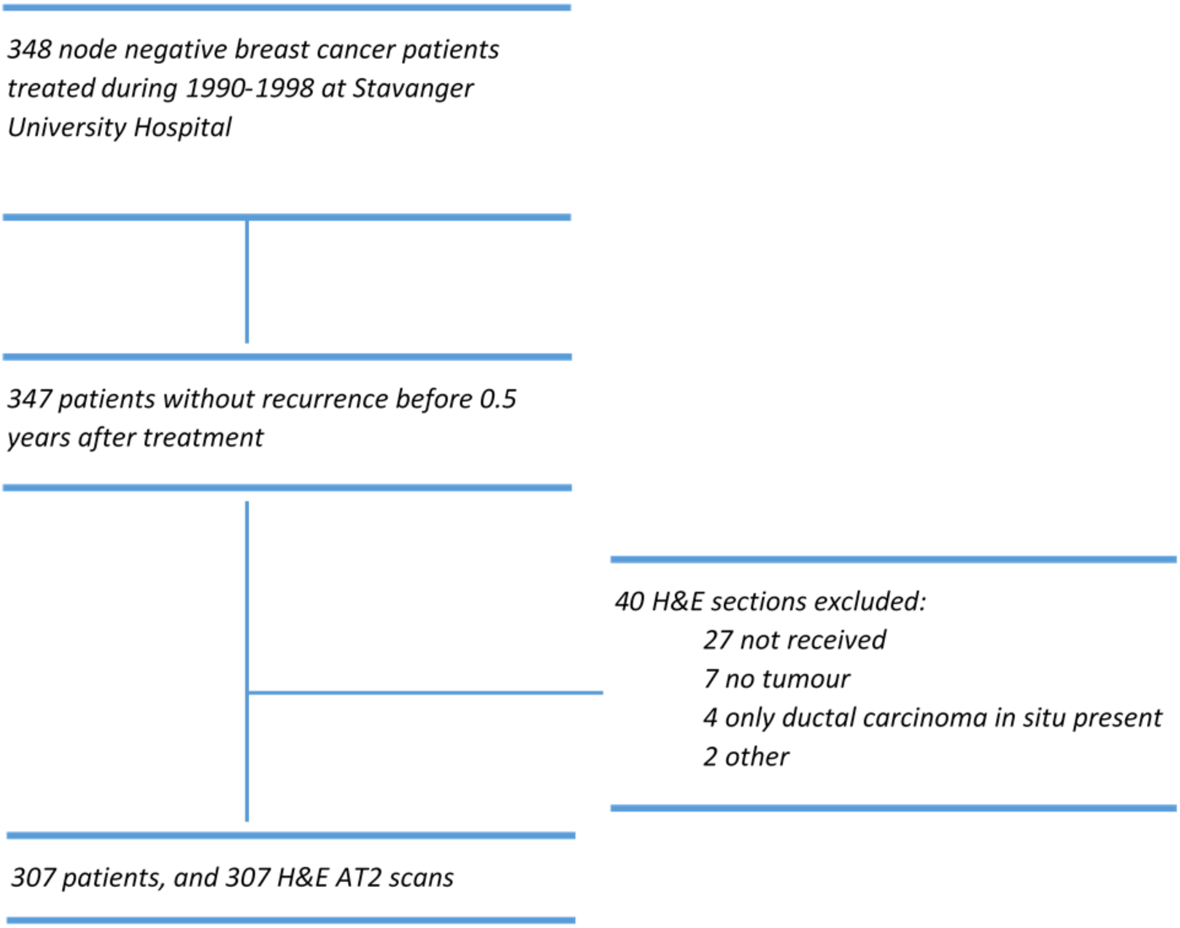

Protocol Figure 5: Inclusions and exclusions of patients and samples in the breast cancer cohort 1990-1998 from Stavanger University Hospital

Parameters to include in multivariable analyses:

- Automatic mitotic figure count per mm^2
- Age at surgery (as continuous variable)
- Oestrogen receptor status
- Progesteron receptor status
- HER2 status
- Nottingham grade
- Nuclear atypia
- Tumour size

Endpoint in uni- and multivariable survival analyses: time to distant recurrence, as used e.g. in [6-9].

Breast cancer cohort 2000-2004 from Stavanger University Hospital

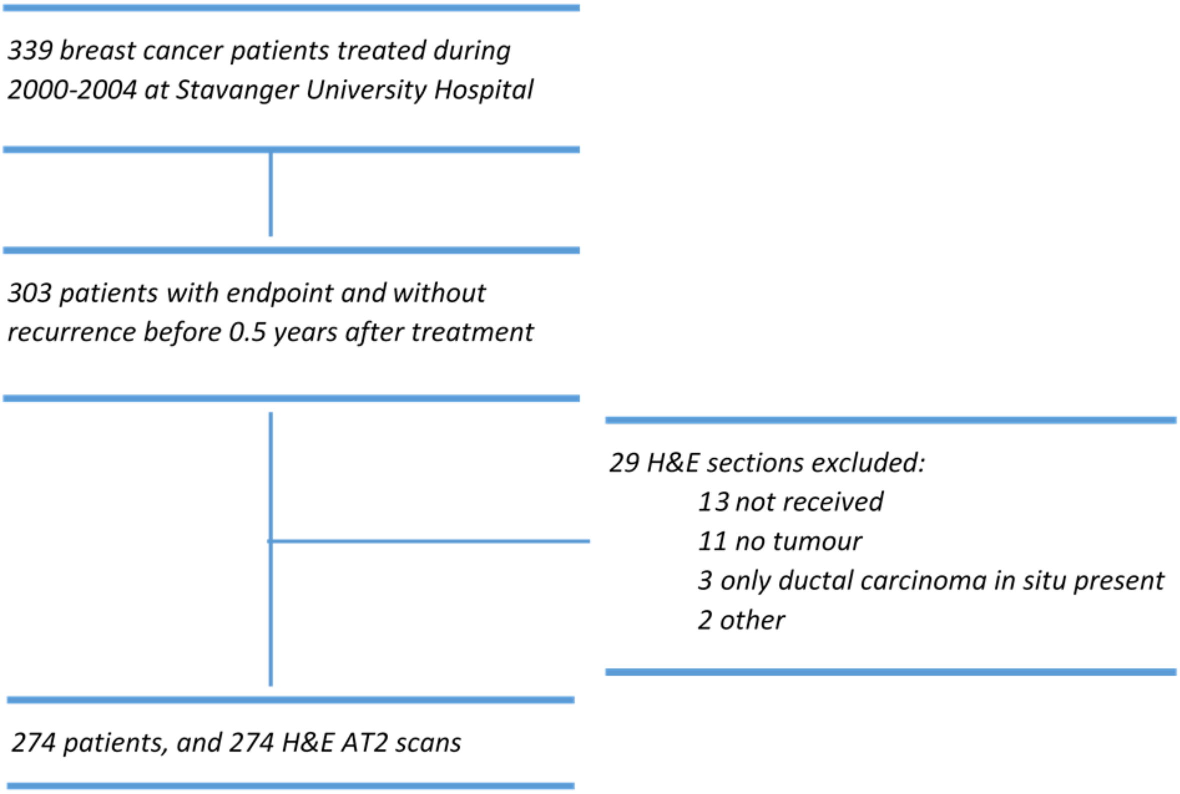

Protocol Figure 6: Inclusions and exclusions of patients and samples in the breast cancer cohort 2000-2004 from Stavanger University Hospital

Parameters to include in multivariable analyses:

- Automatic mitotic figure count per mm^2
- Age at surgery (as continuous variable)
- Oestrogen receptor status
- Progesteron receptor status
- Nottingham grade
- Nuclear atypia
- Tumour size
- Lymph node status

Endpoint in uni- and multivariable survival analyses: time to distant recurrence, as used e.g. in Egeland et al. [10].

Endometrial cancer cohort from Oslo University Hospital

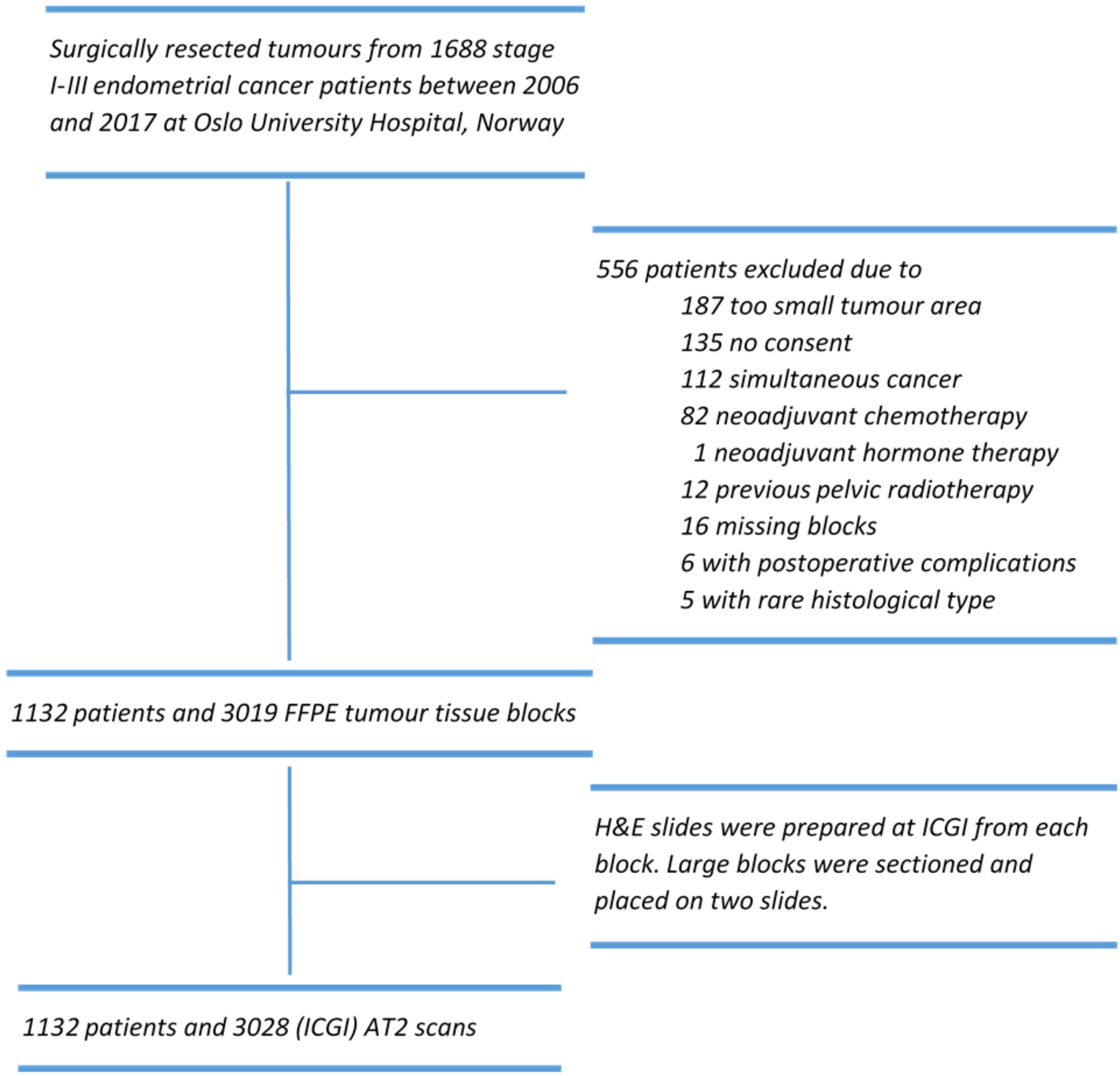

Protocol Figure 7: Inclusions and exclusions of patients and samples in the endometrial cancer cohort from Oslo University Hospital

Parameters to include in multivariable analyses:

- Automatic mitotic figure count per mm^2
- Age (<60 vs >=60)
- FIGO stage
- Histology and differentiation
    - Endometrioid and grade 1
    - Endometrioid and grade 2
    - Endometrioid and grade 3
    - Mixed without c/s and grade 1

    - Mixed without c/s and grade 2
    - Mixed without c/s and grade 3
    - Mucinous
    - Clear cell
    - Serous
    - Mixed with c/s
    - Undifferentiated carcinoma
    - Unclassifiable carcinoma
    - Neuroendocrine
    - Karcinosarcoma
- LVSI
- Adjuvant treatment

Primary endpoint in uni- and multivariable survival analyses: time to recurrence, as defined in [11].

Lung cancer cohort from Oslo University Hospital

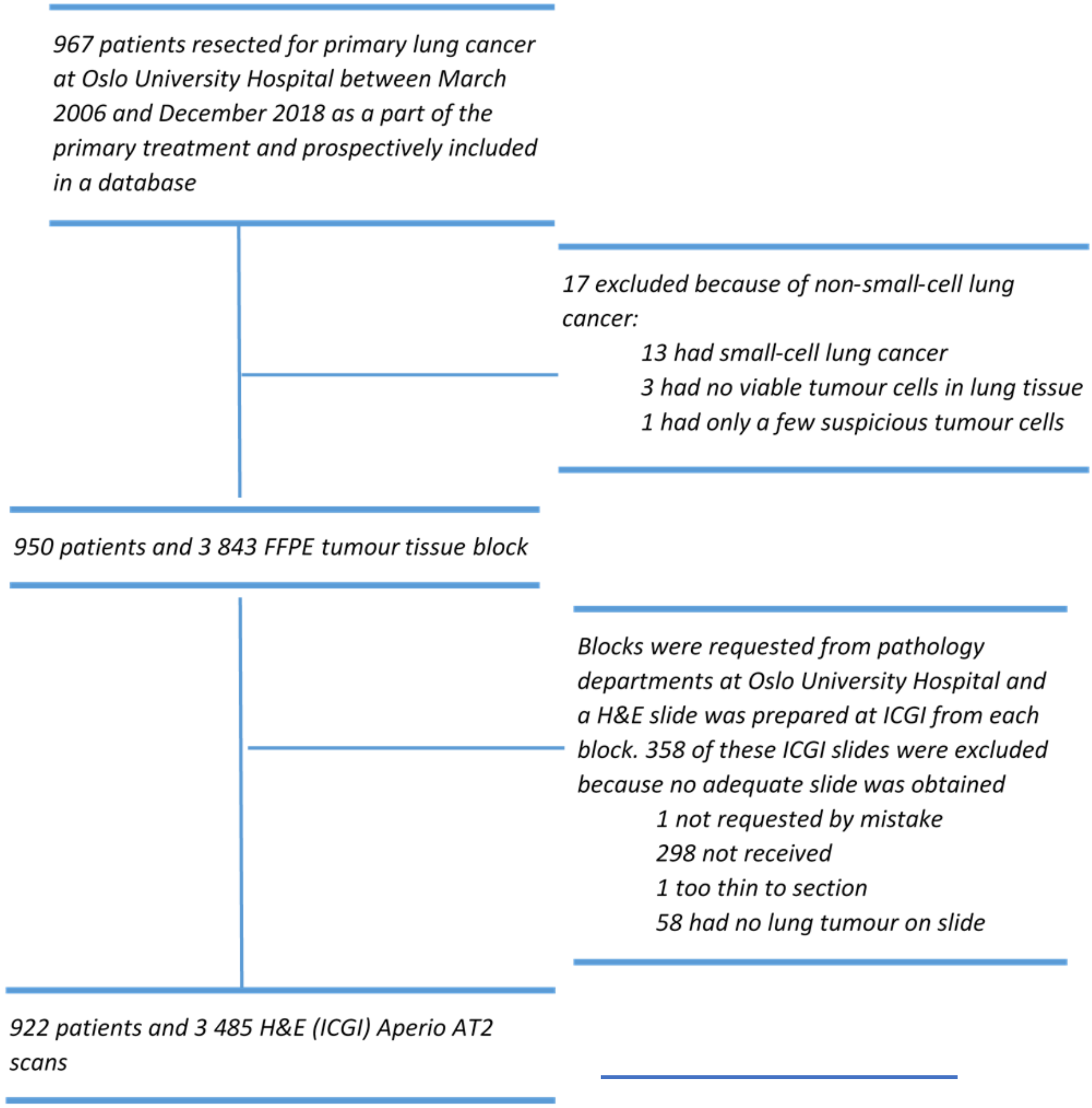

Protocol Figure 8: Inclusions and exclusions of patients and samples in the lung cancer cohort from Oslo University Hospital

Parameters to include in multivariable analyses:

- Automatic mitotic figure count per mm^2
- Age
- Gender
- Smoking status
- Pathological stage
- ECOG performance status
- Histological type

Primary endpoint in uni- and multivariable survival analyses: cancer-specific survival.

Lung cancer cohort from University Hospital of North Norway and Nordland Hospital

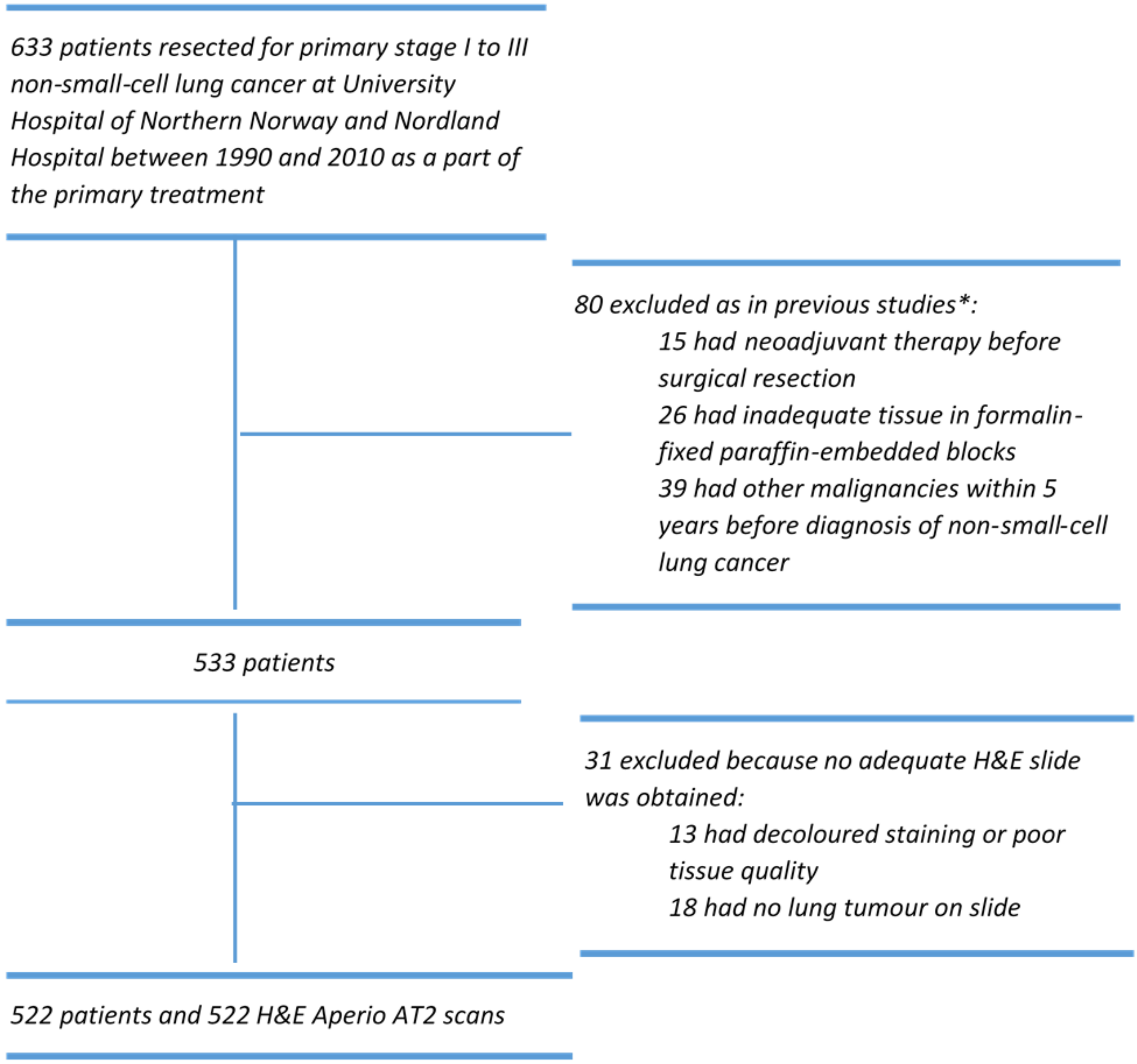

Protocol Figure 9: Inclusions and exclusions of patients and samples in the lung cancer cohort from University Hospital of North Norway and Nordland Hospita

**Replicates the exclusions in previous studies.[12, 13]*

Parameters to include in multivariable analyses:

- Automatic mitotic figure count per mm^2
- Age
- Gender
- Smoking status
- Pathological stage
- ECOG performance status
- Histological type

Primary endpoint in uni- and multivariable survival analyses: cancer-specific survival.

Prostate cancer cohort 1 from Oslo University Hospital

317 patients diagnosed with prostate cancer who underwent radical prostatectomy (RP) between 1987-2005 at the Norwegian Radium Hospital, Oslo. Neoadjuvant therapy was not given to any of the included patients. Adjuvant therapy was started in cases of postoperative PSA elevation and/or metastatic disease

10 patients were excluded due to following

- *1 preoperative therapy*
- *1 death from postoperative complications*
- *1 loss to follow-up*
- *7 tumour material unavailable*

307 eligible patients

A subset of 254 patients were selected with three available tumor-containing blocks, based on the highest Gleason sum and/or previously assessed non-diploid DNA ploidy status

254 patients, 762 FFPE tumour tissue blocks

H&E slides were prepared at ICGI from each block. 12 ICGI slides were excluded:

- *12 No tumor*

253 patients, 750 H&E (ICGI) Aperio AT2 scans

Protocol Figure 10: Inclusions and exclusions of patients and samples in prostate cancer cohort 1 from Oslo University Hospital

Parameters to include in multivariable analyses:

- Automatic mitotic figure count per mm^2
- Age at surgery (as continuous variable)
- Preoperative PSA, log2-transformed
- Gleason score (<=6, 3+4, 4+3, 8, >=9)
- Extracapsular extension (no, yes)
- Seminval vesicle invasion (no, yes)

- Surgical margins (no (free), yes (not free))
- Lymph node invasion (no, yes)

Primary endpoint in uni- and multivariable survival analyses: time to recurrence, as defined in [11]. Time was calculated from surgery to date of endpoint or loss to follow-up.

Prostate cancer cohort 2 from Oslo University Hospital

287 patients diagnosed with prostate cancer who underwent radical prostatectomy (RP) between 2001-2006 at the Norwegian Radium Hospital, Oslo.
2 patients received neoadjuvant therapy and 16 received adjuvant hormonal or radiotherapy within first six months after surgery.

28 patients were excluded due to the following
21 missing consent
4 missing or less than six weeks follow-up
3 no tumour material available

259 eligible patients

Three tumour blocks per patient were selected for all patients; Highest Gleason score, largest tumour area and random block with tumour area > 5 mm^2

259 patients, 777 FFPE tumour tissue blocks

H&E slides were prepared at ICGI from each block.

259 patients, 777 H&E (ICGI) Aperio AT2 scans

Protocol Figure 11: Inclusions and exclusions of patients and samples in prostate cancer cohort 2 from Oslo University Hospital

Parameters to include in multivariable analyses:

- Automatic mitotic figure count per mm^2
- Age at surgery (as continuous variable)
- Preoperative PSA, log2-transformed
- Gleason score (<=6, 3+4, 4+3, 8, >=9)
- Extracapsular extension (no, yes)
- Seminval vesicle invasion (no, yes)
- Surgical margins (no (free), yes (not free))
- Lymph node invasion (no, yes)

Primary endpoint in uni- and multivariable survival analyses: biochemical recurrence, defined as the first PSA>=0.4ng/ml during follow-up. Time was calculated from surgery to date of endpoint or loss to follow-up. PSA measurements within 6 weeks after surgery were not considered when identifying the endpoint.

Prostate cancer cohort 3 from Vestfold Hospital Trust

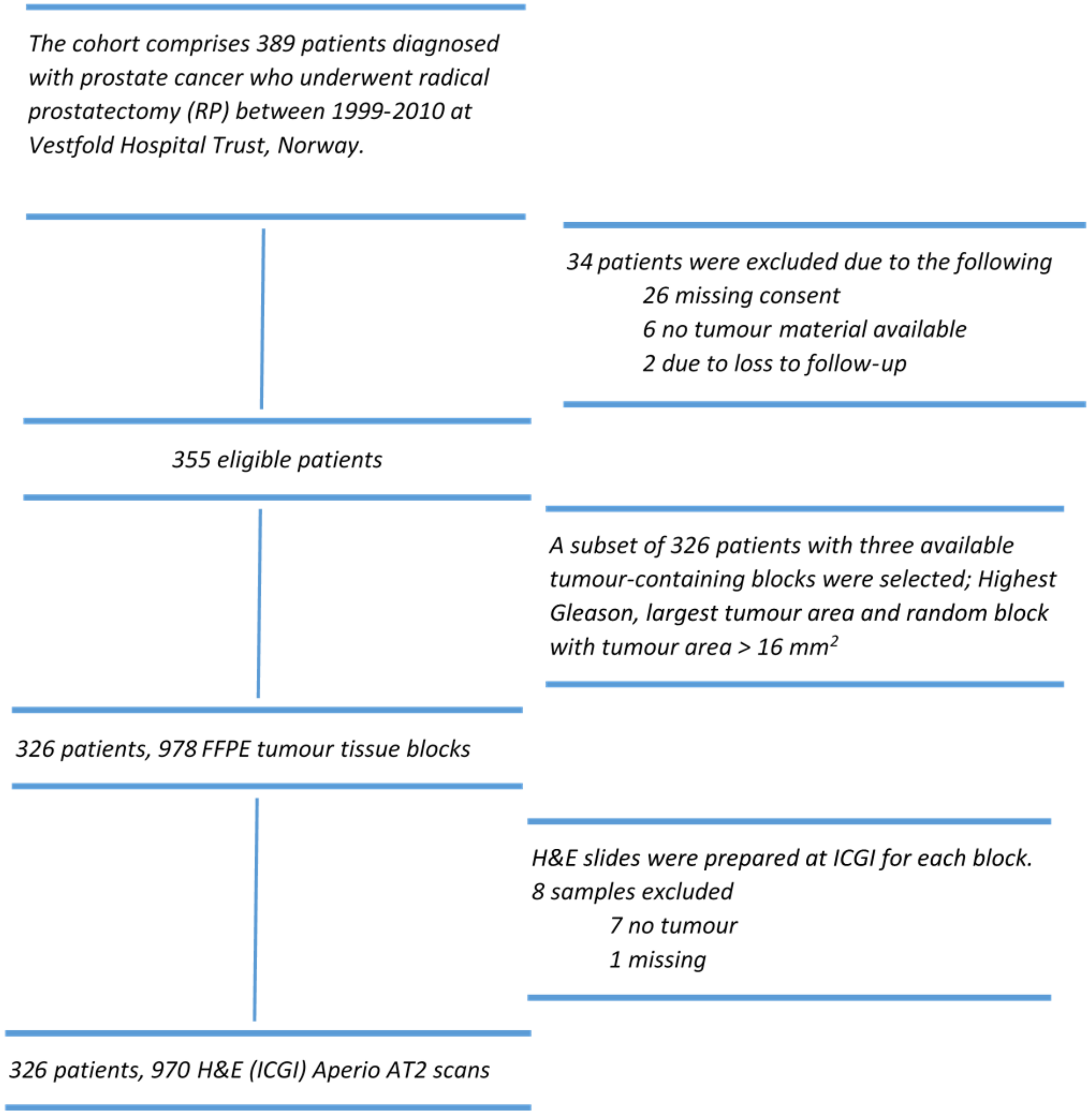

Protocol Figure 12: Inclusions and exclusions of patients and samples in the prostate cancer cohort from Vestfold Hospital Trust

Parameters to include in multivariable analyses:

- Automatic mitotic figure count per mm^2
- Age at surgery (as continuous variable)
- Preoperative PSA, log2-transformed
- Gleason score (<=6, 3+4, 4+3, 8, >=9)
- Extracapsular extension (no, yes)
- Seminval vesicle invasion (no, yes)
- Surgical margins (no (free), yes (not free))
- Lymph node invasion (no, yes)

Primary endpoint in uni- and multivariable survival analyses: biochemical recurrence, defined as the first PSA>=0.4ng/ml during follow-up. Time was calculated from surgery to date of endpoint or loss to follow-up. PSA measurements within 6 weeks after surgery were not considered when identifying the endpoint.

Prostate cancer cohort 4 from University Hospital of North Norway

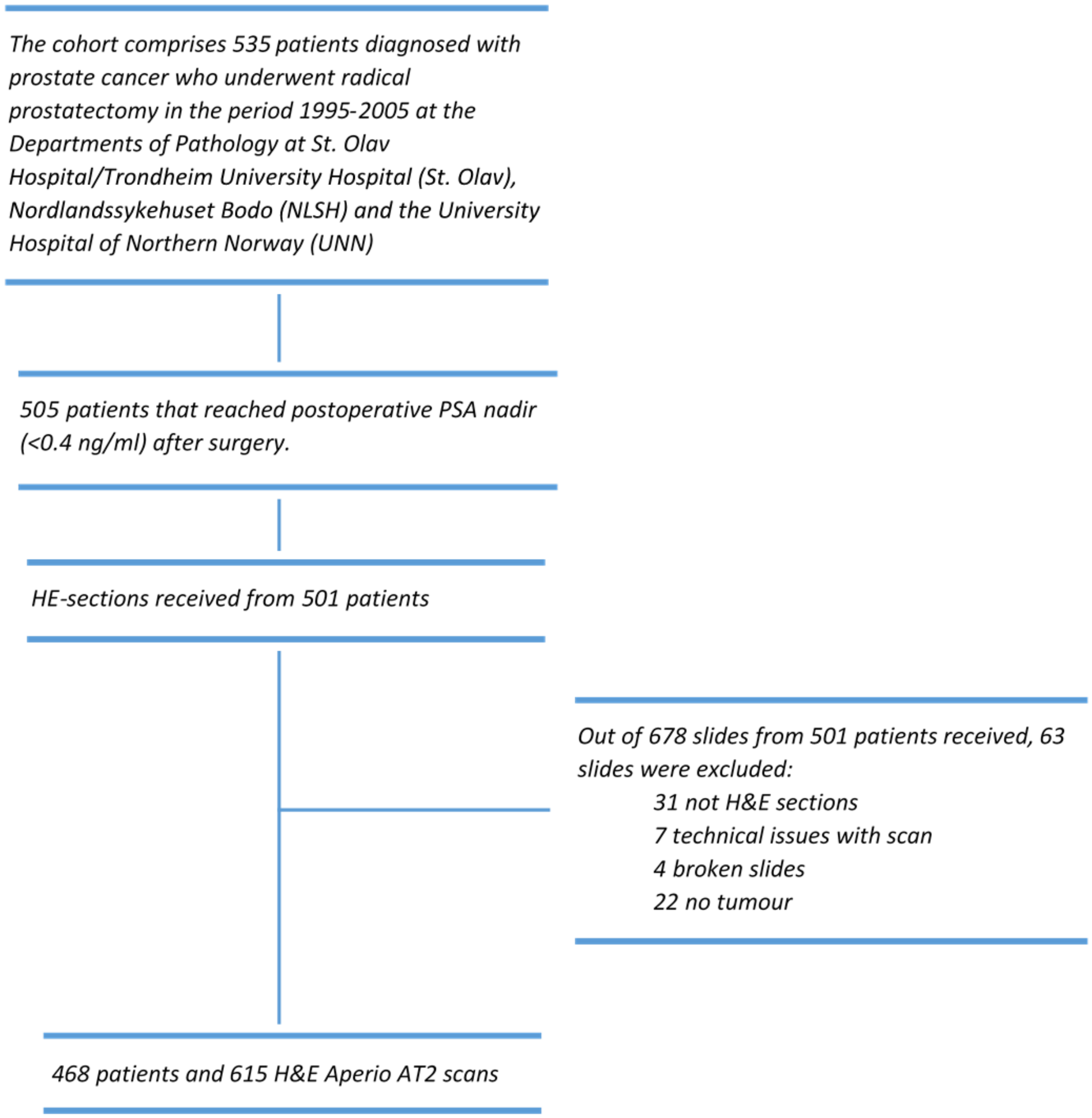

Protocol Figure 13: Inclusions and exclusions of patients and samples in the prostate cancer cohort from St. Olav Hospital/Trondheim University Hospital (St. Olav), Nordlandssykehuset Bodo (NLSH) and the University Hospital of Northern Norway (UNN)

Parameters to include in multivariable analyses:

- Automatic mitotic figure count per mm^2
- Age at surgery (as continuous variable)
- Preoperative PSA, log2-transformed
- Gleason score (<=6, 3+4, 4+3, 8, >=9)
- Pathological T-stage (pT)
- Surgical margins (no (free), yes (not free))
- Lymph node invasion (no, yes)

Primary endpoint in uni- and multivariable survival analyses: biochemical recurrence, defined as the first PSA>=0.4ng/ml and rising during follow-up, used in [14]. Time was calculated from surgery to date of endpoint or loss to follow-up. Patients who did not reach a PSA level of 0.4 ng/ml after surgery were not included in analyses.

Bladder cancer cohort from Stavanger University Hospital

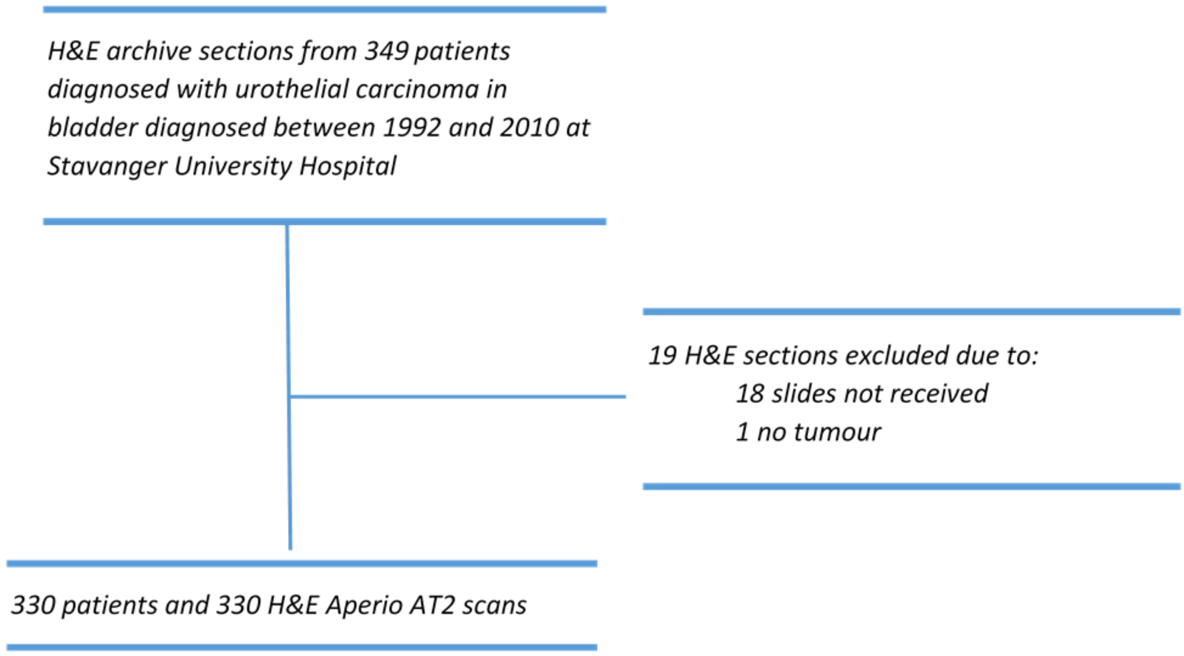

Protocol Figure 14: Inclusions and exclusions of patients and samples in the bladder cancer cohort from Stavanger University Hospital

Parameters to include in multivariable analyses:

- Automatic mitotic figure count per mm^2
- Age at diagnosis (as continuous variable)
- Gender (female/male)
- WHO 2004 grade (low grade/high grade)
- Multifocality (no/yes)
- CIS (no/yes)

Primary endpoint in uni- and multivariable survival analyses: time to stage progression, as e.g. used in [15, 16].

Liver metastases cohort from Liverpool University Hospitals

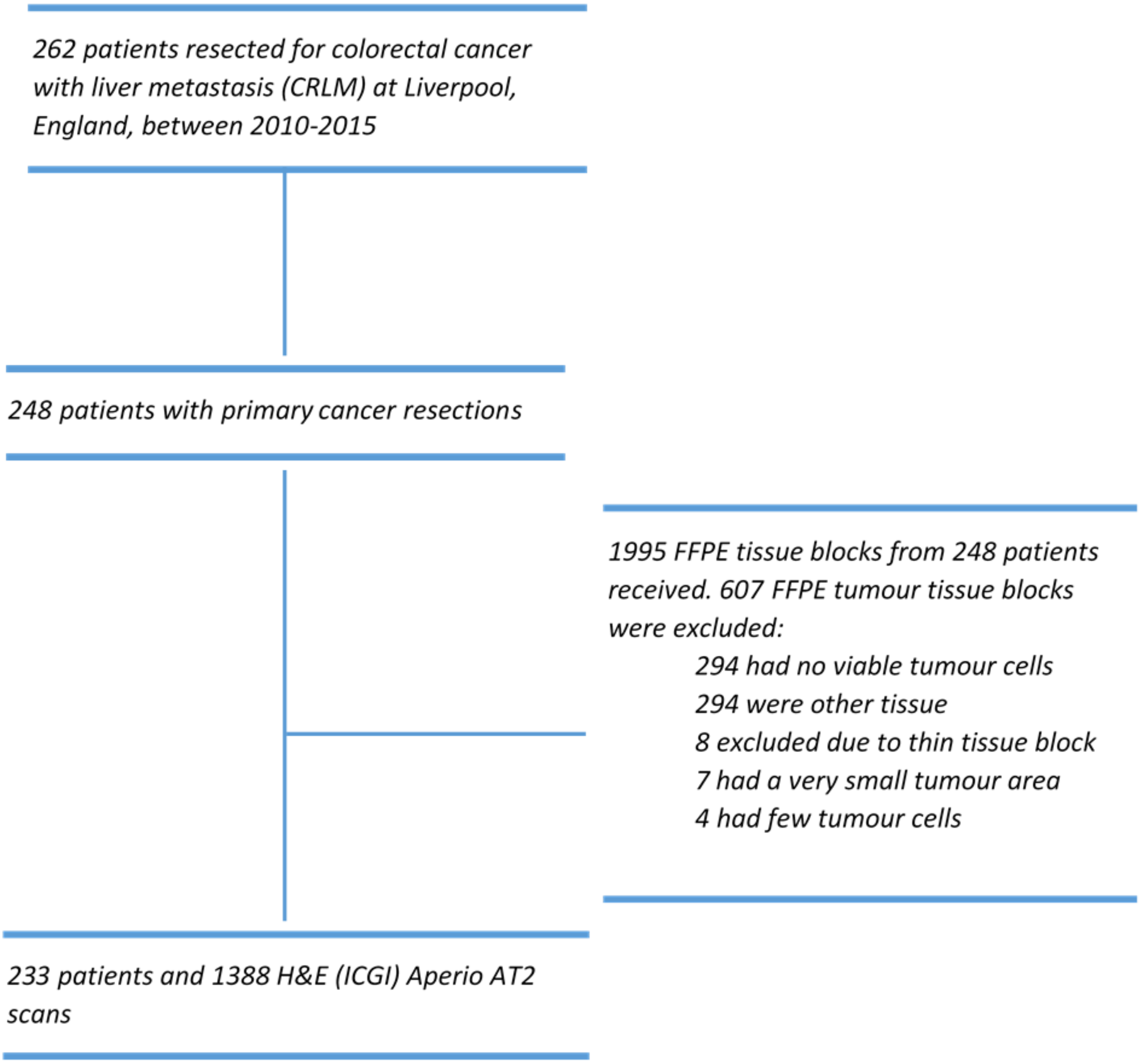

Protocol Figure 15: Inclusions and exclusions of patients and samples in the liver metastases cohort from Liverpool University Hospitals

Parameters to include in multivariable analyses:

- Automatic mitotic figure count per mm^2
- Age
- Gender
- Synchronous/metachronous
- Size of largest lesion (<=50mm vs >50mm)
- Number of metastases (1 vs >1 lesions)
- Pathological T stage of primary tumour
- Pathological N stage of primary tumour
- Location of primary tumour (left vs right)
- Extra hepatic disease
- Neoadjuvant therapy

- Adjuvant therapy

Primary endpoint in uni- and multivariable survival analyses: overall survival, calculated from date of last surgery (either liver or colorectal) for patients with synchronous disease, and date of liver surgery for those with metachronous disease.

Colorectal cancer cohort from Cheltenham General Hospital

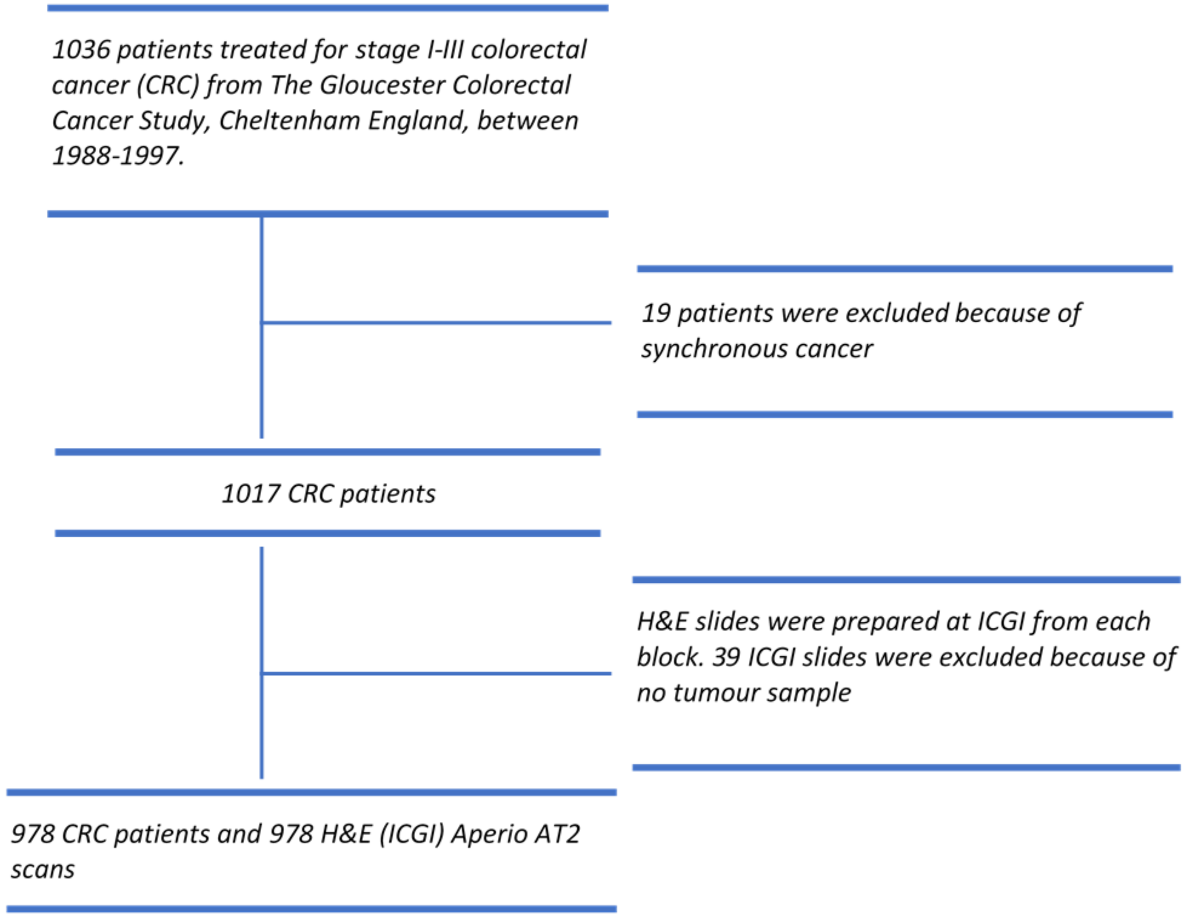

Protocol Figure 16: Inclusions and exclusions of patients and samples in the colorectal cancer cohort from Cheltenham General Hospital

Parameters to include in multivariable analyses:

- Automatic mitotic figure count per mm^2
- Age
- Pathological T stage of primary tumour
- Pathological N stage of primary tumour
- Tumour location (left/right/rectum)

Primary endpoint in uni- and multivariable survival analyses: cancer-specific survival.

Colorectal cancer cohort from the Quasar2 trial

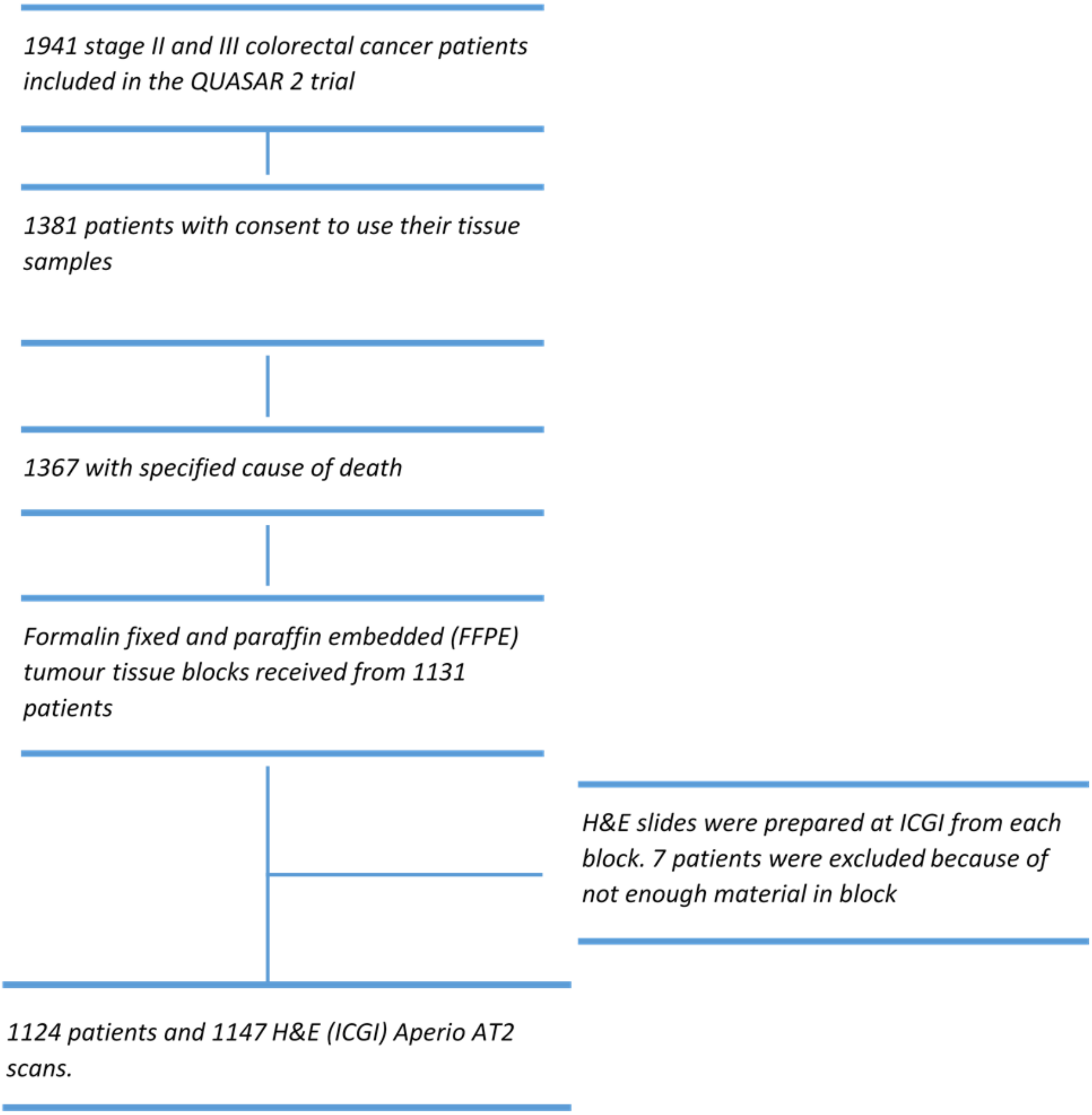

Protocol Figure 17: Inclusions and exclusions of patients and samples in the QUASAR 2 colorectal cancer cohort[17]

Parameters to include in multivariable analyses:

- Automatic mitotic figure count per mm^2
- Age
- Pathological T stage of primary tumour
- Pathological N stage of primary tumour
- Tumour location (left/right/rectum)

Primary endpoint in uni- and multivariable survival analyses: cancer-specific survival.

Analyses

Primary analysis

The primary analysis aims to evaluate the prognostic impact of the deep learning-based method for mitotic figure counting in 13 independent cancer patient cohorts. The prognostic impact of the method will be assessed using univariable Cox proportional hazard regression with the log-transformed detected number of mitotic figures per mm^2 as a continuous variable with calculation of hazard ratio (HR) with corresponding 95% confidence interval (CI) and p-value from the Wald test in each cancer patient cohort using significance level 0.05. The log-transformation of the predicted number of mitotic figures per mm^2 will be log (number-of-mitotic-figures-per-mm^2 + 1), using the binary logarithm. Primary endpoints are specified for each cancer patient cohort. The tumour region in each scanned HE-stained tissue section is manually annotated before the deep-learning based method for mitotic figure counting is applied. The method returns the predicted number of mitotic figures per mm^2 in the annotated tumour region. For patients with multiple scanned tissue sections, the total mitotic figure count in all tissue sections divided by the total annotated area (in mm^2) is reported.

Secondary analyses

Analyses predefined before evaluation in the validation cohorts:

1. Multivariable Cox proportional hazard regression analysis with log-transformed detected mitotic figure counts per mm^2 together with established prognostic markers in each cancer patient cohort. Variables to be included are specified in the description of each cancer patient cohort. Exclude patients with missing value for any of the included variables. Calculate HR with 95% CI and corresponding p-values using the same endpoints as in the primary analysis.
2. Categorise the mitotic figure counts per mm^2 using quartiles calculated in each individual cancer patient cohort. Calculate HR with 95% CI and corresponding p-value from the Mantel-Cox log-rank test for the categorised mitotic figure count using the same endpoints as in the primary analysis.
3. Univariable analysis similar to the primary analysis with other endpoints that are available in the respective validation cohorts.

References

1. He, K., Gkioxari, G., Dollár, P. & Girshick, R. (2017). Mask R-CNN. Paper presented at the *2017 IEEE International Conference on Computer Vision (ICCV)*.
2. Russakovsky, O., Deng, J., Su, H., Krause, J., Satheesh, S., Ma, S., Huang, Z., Karpathy, A., Khosla, A. & Bernstein, M. (2015) Imagenet large scale visual recognition challenge, *International journal of computer vision.* **115**, 211-252.
3. Macenko, M., Niethammer, M., Marron, J. S., Borland, D., Woosley, J. T., Xiaojun, G., Schmitt, C. & Thomas, N. E. (2009). A method for normalizing histology slides for quantitative analysis. Paper presented at the *2009 IEEE International Symposium on Biomedical Imaging: From Nano to Macro*.
4. Abeler, V. M., Royne, O., Thoresen, S., Danielsen, H. E., Nesland, J. M. & Kristensen, G. B. (2009) Uterine sarcomas in Norway. A histopathological and prognostic survey of a total population from 1970 to 2000 including 419 patients, *Histopathology.* **54**, 355-64.
5. Harrell, F. E., Jr., Califf, R. M., Pryor, D. B., Lee, K. L. & Rosati, R. A. (1982) Evaluating the yield of medical tests, *JAMA.* **247**, 2543-6.
6. Skaland, I., Janssen, E. A., Gudlaugsson, E., Hui Ru Guo, L. & Baak, J. P. (2009) The prognostic value of the proliferation marker phosphohistone H3 (PPH3) in luminal, basal-like and triple negative phenotype invasive lymph node-negative breast cancer, *Cell Oncol.* **31**, 261-71.
7. Skaland, I., Janssen, E. A., Gudlaugsson, E., Klos, J., Kjellevold, K. H., Søiland, H. & Baak, J. P. (2009) Validating the prognostic value of proliferation measured by Phosphohistone H3 (PPH3) in invasive lymph node-negative breast cancer patients less than 71 years of age, *Breast Cancer Res Treat.* **114**, 39-45.
8. Jonsdottir, K., Zhang, H., Jhagroe, D., Skaland, I., Slewa, A., Björkblom, B., Coffey, E. T., Gudlaugsson, E., Smaaland, R., Janssen, E. A. & Baak, J. P. (2012) The prognostic value of MARCKS-like 1 in lymph node-negative breast cancer, *Breast Cancer Res Treat.* **135**, 381-90.
9. Jonsdottir, K., Janssen, S. R., Da Rosa, F. C., Gudlaugsson, E., Skaland, I., Baak, J. P. & Janssen, E. A. (2012) Validation of expression patterns for nine miRNAs in 204 lymph-node negative breast cancers, *PLoS One.* **7**, e48692.
10. Egeland, N. G., Austdal, M., van Diermen-Hidle, B., Rewcastle, E., Gudlaugsson, E. G., Baak, J. P. A., Skaland, I., Janssen, E. A. M. & Jonsdottir, K. (2019) Validation study of MARCKSL1 as a prognostic factor in lymph node-negative breast cancer patients, *PLoS One.* **14**, e0212527.
11. Punt, C. J., Buyse, M., Kohne, C. H., Hohenberger, P., Labianca, R., Schmoll, H. J., Pahlman, L., Sobrero, A. & Douillard, J. Y. (2007) Endpoints in adjuvant treatment trials: a systematic review of the literature in colon cancer and proposed definitions for future trials, *J Natl Cancer Inst.* **99**, 998-1003.
12. Hald, S. M., Rakaee, M., Martinez, I., Richardsen, E., Al-Saad, S., Paulsen, E. E., Blix, E. S., Kilvaer, T., Andersen, S., Busund, L. T., Bremnes, R. M. & Donnem, T. (2018) LAG-3 in Non-Small-cell Lung Cancer: Expression in Primary Tumors and Metastatic Lymph Nodes Is Associated With Improved Survival, *Clin Lung Cancer.* **19**, 249-259.e2.
13. Rakaee, M., Busund, L. R., Jamaly, S., Paulsen, E. E., Richardsen, E., Andersen, S., Al-Saad, S., Bremnes, R. M., Donnem, T. & Kilvaer, T. K. (2019) Prognostic Value of Macrophage Phenotypes in Resectable Non-Small Cell Lung Cancer Assessed by Multiplex Immunohistochemistry, *Neoplasia.* **21**, 282-293.
14. Andersen, S., Richardsen, E., Nordby, Y., Ness, N., Størkersen, O., Al-Shibli, K., Donnem, T., Bertilsson, H., Busund, L. T., Angelsen, A. & Bremnes, R. M. (2014) Disease-specific outcomes of radical prostatectomies in Northern Norway; a case for the impact of perineural infiltration and postoperative PSA-doubling time, *BMC Urol.* **14**, 49.
15. Kvikstad, V., Mangrud, O. M., Gudlaugsson, E., Dalen, I., Espeland, H., Baak, J. P. A. & Janssen, E. A. M. (2019) Prognostic value and reproducibility of different microscopic characteristics in the WHO grading systems for pTa and pT1 urinary bladder urothelial carcinomas, *Diagn Pathol.* **14**, 90.
16. Lillesand, M., Kvikstad, V., Mangrud, O. M., Gudlaugsson, E., van Diermen-Hidle, B., Skaland, I., Baak, J. P. A. & Janssen, E. A. M. (2020) Mitotic activity index and CD25+ lymphocytes predict risk of stage progression in non-muscle invasive bladder cancer, *PLoS One.* **15**, e0233676.
17. Kerr, R. S., Love, S., Segelov, E., Johnstone, E., Falcon, B., Hewett, P., Weaver, A., Church, D., Scudder, C., Pearson, S., Julier, P., Pezzella, F., Tomlinson, I., Domingo, E. & Kerr, D. J. (2016) Adjuvant capecitabine plus bevacizumab versus capecitabine alone in patients with colorectal cancer (QUASAR 2): an open-label, randomised phase 3 trial, *Lancet Oncol.* **17**, 1543-1557.

Protocol Amendment 27.01.2026

Following feedback in the review process, we made the following three changes to the protocol:

1. The protocol figure legends were relabeled as Protocol Figure.
2. Scale bars were added to Protocol Figures 2 and 3.
3. References were reformatted to the style of FEBS Open Bio.
